# Mobile primary health care clinics for Indigenous populations in Australia, Canada, New Zealand and the United States: a systematic scoping review

**DOI:** 10.1186/s12939-020-01306-0

**Published:** 2020-11-09

**Authors:** Hannah Beks, Geraldine Ewing, James A. Charles, Fiona Mitchell, Yin Paradies, Robyn A. Clark, Vincent L. Versace

**Affiliations:** 1 School of Medicine, Deakin University, Geelong, Victoria Australia; 2 National Indigenous Knowledges Education Research Innovation (NIKERI) Institute, Deakin University, Geelong, Victoria Australia; 3 Faculty of Arts and Education, Deakin University, Burwood, Victoria Australia; 4 College of Nursing and Health Sciences, Flinders University, Adelaide, South Australia

**Keywords:** Global health, Health services, Indigenous health, Mobile health clinics, Primary health care

## Abstract

**Background:**

Mobile clinics have been used to deliver primary health care to populations that otherwise experience difficulty in accessing services. Indigenous populations in Australia, Canada, New Zealand, and the United States experience greater health inequities than non-Indigenous populations. There is increasing support for Indigenous-governed and culturally accessible primary health care services which meet the needs of Indigenous populations. There is some support for primary health care mobile clinics implemented specifically for Indigenous populations to improve health service accessibility. The purpose of this review is to scope the literature for evidence of mobile primary health care clinics implemented specifically for Indigenous populations in Australia, Canada, New Zealand, and the United States.

**Methods:**

This review was undertaken using the Joanna Brigg Institute (JBI) scoping review methodology. Review objectives, inclusion criteria and methods were specified in advance and documented in a published protocol. The search included five academic databases and an extensive search of the grey literature.

**Results:**

The search resulted in 1350 unique citations, with 91 of these citations retrieved from the grey literature and targeted organisational websites. Title, abstract and full-text screening was conducted independently by two reviewers, with 123 citations undergoing full text review. Of these, 39 citations discussing 25 mobile clinics, met the inclusion criteria. An additional 14 citations were snowballed from a review of the reference lists of included citations. Of these 25 mobile clinics, the majority were implemented in Australia (*n* = 14), followed by United States (*n* = 6) and Canada (*n* = 5). No primary health mobile clinics specifically for Indigenous people in New Zealand were retrieved. There was a pattern of declining locations serviced by mobile clinics with an increasing population. Furthermore, only 13 mobile clinics had some form of evaluation.

**Conclusions:**

This review identifies geographical gaps in the implementation of primary health care mobile clinics for Indigenous populations in Australia, Canada, New Zealand, and the United States. There is a paucity of evaluations supporting the use of mobile clinics for Indigenous populations and a need for organisations implementing mobile clinics specifically for Indigenous populations to share their experiences. Engaging with the perspectives of Indigenous people accessing mobile clinic services is imperative to future evaluations.

**Registration:**

The protocol for this review has been peer-reviewed and published in JBI Evidence Synthesis (doi: 10.11124/JBISRIR-D-19-00057).

**Supplementary Information:**

**Supplementary information** accompanies this paper at10.1186/s12939-020-01306-0.

## Background

Accessible primary health care is an inherent human right for all populations, as stipulated by the Declaration of Alma-Ata (1978) [1]. Primary health care encompasses early interventions delivered by general practitioners, nurses and allied health professionals such as health promotion, screening for disease and health education for disease prevention [1, 2]. Evidence supports the effectiveness of primary health care services in improving the management of chronic disease and addressing risk factors for developing chronic disease, across a range of contexts [3–6]. However, primary health care services are not always accessible for all populations. This is the case for Indigenous populations in Australia, Canada, New Zealand and the United States, who often experience racism, cultural, transport and financial barriers when accessing health services [7–10].

The multi-dimensional nature of health care access is well documented which includes the availability, accessibility, accommodation, affordability, acceptability and awareness of health care services [11, 12]. For Indigenous people, an important component of health care access is the provision of culturally safe and holistic health care by a trusted health professional who respects their values, traditions and customs [13–15]. Across the globe, Indigenous populations are culturally and linguistically diverse, with differing environmental contexts (e.g. climates, connections to land and waterways), cultural practices (e.g. lore, customs, spiritual beliefs) and cultural identities (e.g. kinship ties, ancestors) [16]. In modern states with a history of invading Indigenous lands through the process of colonization (e.g. Australia, Canada, New Zealand and United States), there are numerous Indigenous nations, tribes and clans, all with unique cultural identities, histories and languages [16]. However, there are similarities in the experience of colonialization for Indigenous people (e.g. racism, violence, experience of European communicable diseases and loss of land), particularly in Australia, Canada, New Zealand, and the United States, which has led to enduring inequity [7, 17, 18].

To redress health inequities for Indigenous populations, including the burden of chronic disease and high mortality rate compared to non-Indigenous populations [18], culturally safe models of health care are needed which improve the accessibility of primary health care services [19]. Evidence supports that a greater participation of Indigenous people in their health care leads to better health outcomes [20, 21]. Therefore, Indigenous-governed health care services are inherent to the provision of culturally accessible health care [22]. In Australia, over 140 Aboriginal Community-Controlled Health Services (ACCHOs) provide primary health care services to Aboriginal and Torres Strait Islander people [23]. Internationally, evidence supports the important contribution of Indigenous-governed health organisations in providing culturally safe and accessible primary health care for Indigenous populations [24–27].

Mobile clinics implemented specifically for Indigenous populations and governed by Indigenous health organisations, may be one way to improve the accessibility of culturally safe primary health care for Indigenous populations. It is known that mobile clinics are able to deliver health care to populations experiencing health inequity, particularly in countries where health care can be otherwise inaccessible due to transport, financial or cultural barriers [28–30]. In the United States, there has been an upward surge in the implementation of mobile clinics, particularly of mobile clinics delivering primary health care services [31, 32]. The support for mobile clinics in providing flexible and safe health care to vulnerable people has gained traction with the recent COVID-19 pandemic [33]. In other countries, mobile clinics have also been implemented with the purpose of screening for communicable and non-communicable diseases [34–36] and providing disaster relief [37, 38]. Some research supports the potential for mobile clinics to be a cost-effective model of health care and improve the management of chronic disease [29, 39].

There is also some evidence of mobile clinics being implemented specifically for Indigenous populations, either by an Indigenous health organization [40] or for a specific disease (e.g. diabetes) [41] or treatment (e.g. dialysis) [42]. What is not known, is the available evidence regarding the use of primary health care mobile clinics implemented specifically for Indigenous populations in Australia, Canada, New Zealand, and the United States who share a similar history of colonization, discrimination and barriers to accessing primary health care services [7]. This was apparent when undertaking a preliminary search of the literature for evidence around the effectiveness of mobile clinics for Indigenous populations, as part of seeking funding for a mobile clinic to be implemented in an Australian ACCHO. Indeed, it was an absence of evidence that made it difficult to obtain funding for the mobile clinic, justifying the need for a systematic scoping review. It is known that there is a vast body of literature regarding mobile clinics in the United States, yet there is very little focus on Native American, Native Hawaiian, and Alaskan Native populations [32]. A systematic scoping review was conceptualised to synthesise the available evidence regarding the use of primary health care mobile clinics implemented specifically for Indigenous populations in order to identify gaps in the literature and inform future research evaluating mobile clinics for Indigenous populations. Specifically, the review question developed was:

What is the evidence surrounding the use of mobile primary healthcare clinics implemented for Indigenous populations in Australia, Canada, New Zealand, and the United States?

Specific objectives were to: (1) scope the models of primary health care clinics for Indigenous populations (in Australia, Canada, New Zealand, and the United States) as described in the literature, (2) determine geographically where mobile primary health care clinics for Indigenous populations (in Australia, Canada, New Zealand, and the United States) have been implemented and, (3) examine the findings of any evaluations of mobile primary health care clinics for Indigenous populations (in Australia, Canada, New Zealand, and the United States) that have been published in the literature.

## Methods

This systematic scoping review examines the evidence surrounding the use of mobile primary healthcare clinics implemented for Indigenous populations in Australia, Canada, New Zealand, and the United States [43]. This review was conducted in accordance with the Joanna Briggs Institute (JBI) Reviewer’s Manual 2017: Methodology for JBI Scoping Reviews [44]. Search terms were developed using a PCC (Population, Concept, Context) mnemonic. The premise and methods of this review, have been published elsewhere [43]. The Preferred Reporting Items for Systematic Reviews and Meta-analysis extension for scoping reviews checklist (PRISMA-ScR) [45] was adhered to in the reporting of this review (Additional file 1_PRISMA-ScR checklist).

### Search strategy

The JBI three step search process was utilized to develop the search strategy [44]. This involved a preliminary search undertaken in MEDLINE and CINAHL using keywords from the review question. A tailored search was then developed for each information source. For database search strategies, a combination of Boolean operators, truncations and Medical Subject Headings (MeSH) were used (Additional file 2_ Academic database search strategies). Librarian assistance was provided for the development of the Ovid MEDLINE search strategy. Support was also provided in translating the search strategies into other databases. The reference lists of included studies were then searched for additional studies.

Databases searched included: Ovid MEDLINE, CINAHL (EBSCOhost), Embase (Elsevier), Cochrane Database of Systematic Reviews, SocINDEX (EBSCOhost), and INFORMIT.

Multiple platforms were used to search for unpublished studies and grey literature which included: Australian, Canadian, New Zealand, and the United States Indigenous-specific research websites, Indigenous organisational websites, health services and health research websites and open access websites, repositories and catalogues (Additional file 3_Grey Literature sources).

### Inclusion criteria and exclusion criteria

Literature based on the following criteria was considered (Table 1. Inclusion and exclusion criteria).

**Table 1:** Inclusion and exclusion criteria

	Inclusion criteria	Exclusion criteria
**Population**	Indigenous populations across the lifespan (infants, children, adolescents and adults) including; Aboriginal and Torres Strait Islander People (Australia), First Nations, Inuit, and Métis People (Canada), Māori People (New Zealand) and Native American, Native Hawaiian and Alaskan Native People (United States).	No exclusion criteria
**Concept**	Mobile primary health care clinics implemented specifically for Indigenous populations Mobile clinics include a transportable clinic in the form of a van, truck or bus that has been equipped with health equipment	Mobile primary health care clinics implemented for the general population Outreach services delivered by teams of fly in and fly out health professionals Delivery of health care services remotely through mobile technology
**Context**	Mobile primary health care clinics implemented within Australia, Canada, New Zealand and the United States	Mobile clinics delivering only specialist or rehabilitation services Not published in English

No restrictions were placed on the quality or study design used. All types of literature, including media releases, webpages and news articles, were considered. Literature published since 1 January 2006 was considered in order to capture mobile clinics implemented since the ‘United Nations Declaration on the Rights of Indigenous Peoples’ (2007), where a greater international focus on the need to work in partnership with Indigenous populations to improve health outcomes, was established [46].

For consistency, the term ‘Indigenous’ has been used throughout this review to refer to all clans, tribes and communities of Indigenous populations within a global context. We acknowledge the diversity and uniqueness of all Indigenous tribes, clans and nations. No disrespect is intended by the use of this term.

### Study selection and data extraction

Searches for published and unpublished literature were conducted by two researchers (HB and GE). Titles and abstracts retrieved were screened independently by two reviewers (HB and GE). Full text review and data extraction were then undertaken independently by the same two reviewers. For articles not meeting the inclusion criteria, reasons for exclusion were provided. The reference lists of included citations were then screened for additional citations in order to scope for all possible citations meeting the inclusion criteria.

The published data extraction table was used and modified to extract the longitude and latitude coordinates for locations serviced by the included mobile clinics from publicly available information [43]. The coordinates were then imported into ArcGIS ArcMap 10.6.1 (ESRI, CA, USA), a Geographical Information System (GIS), and mapped as point locations. Using a spatial join, the coordinates were linked with an underlying geographical characteristic described either as the Remoteness Structure (Australia) [47], Population Centre and Rural Area Classification 2016 (Canada) [48], or Urban status (United States) [49] to determine the classification of locations serviced by included mobile clinics. It is important to note that each country included in this review has a different rural area classification system. In Australia, Remoteness Structure comprises five categories: Major Cities of Australia, Inner Regional Australia, Outer Regional Australia, Remote Australia, and Very Remote Australia [47]. These classifications offer complete coverage of the Australian continent. Population centers in Canada are described as Small (1000-29,999), Medium (30,000-99,999) or Large (100,000 and over) with all other areas not classified, indicating very low population densities [48]. The urban footprint in the United States (high population density and urban land use) are described as Urban Clusters (2500-49,999) and Urbanised areas (> 50,000) [49]. Like Canada, all other areas are not classified. The spatial data used was based upon each modern state’s most recent census – 2016 for Australia and Canada (next census due 2021), and 2010 for the United States (next census due 2020).

Review findings were developed using a descriptive approach that addressed the review objectives, as per the Joanna Briggs Institute (JBI) Reviewer’s Manual 2017: Methodology for JBI Scoping Reviews [44]. This involved examining the evidence that met the inclusion criteria, providing a summary of citations and synthesising extracted data where possible (e.g. geographical characteristics of location(s) where mobile clinics were implemented).

## Results

**Fig. 1:**
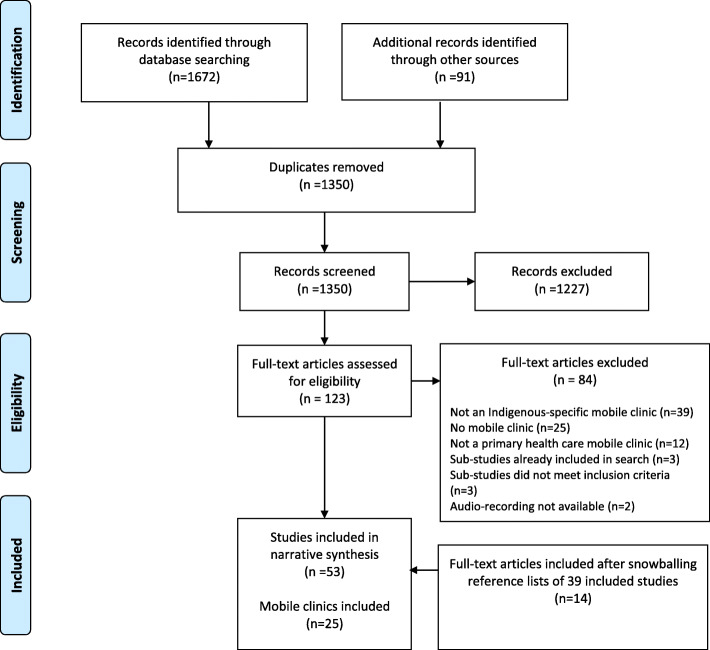
PRISMA diagram of the systematic review process for this review

Database searches yielded 1672 citations. An additional 91 citations were retrieved from an extensive search of the grey literature and targeted organisational websites. A total of 1350 unique title and abstracts were screened, after duplicates were removed. The full texts of 123 citations were screened in accordance with the review criteria, identifying 39 relevant citations (Fig. 1*– PRISMA Flow Diagram*). An additional 14 citations were snow-balled from 39 included citations, resulting in a total of 53 included citations discussing 25 mobile clinics.

Reasons for excluding citations were provided (Additional file 4_Excluded studies) and included: not an Indigenous-specific mobile clinic (*n* = 39), no mobile clinic (*n* = 25), not a primary health care mobile clinic (*n* = 12), sub-studies already included in search (*n* = 3), sub-studies did not meet the inclusion criteria (*n* = 3) and audio-recording not available (*n* = 2).

Information sources of citations meeting the review criteria (*n* = 53) included peer-reviewed journal articles (*n* = 18), conference presentations, papers or posters (*n* = 3), thesis (*n* = 1), independent report (*n* = 1), organisational annual reports or web pages (*n* = 25), and media releases or online news articles (*n* = 5).

### Finding 1: geographical distribution of mobile clinics for Indigenous populations

Of the 25 mobile clinics included (many servicing multiple locations), most were implemented in Australia (*n* = 14), followed by the United States (*n* = 6) and Canada (*n* = 5). No primary health care clinics implemented specifically for Māori populations in New Zealand, were retrieved from the search (Table 2).

**Table 2:** Included mobile primary health care clinics implemented for Indigenous populations

Mobile clinic name	Citation	Year of implementation	Service provider	Country	State/Province
Health E Screen 4 Kids	ABC 2008 [50] Elliot et al. 2010 [51] Nguyen et al. 2015 [52] Smith et al. 2013 [53] Smith et al. 2015 [54] Smith et al. 2012 [55]	2009	University of Queensland	Australia	Queensland
Bega Garnbirringu mobile clinic	Alcohol and Other Drugs Knowledge Centre 2018 [56] Bega Garnbirringu Health Service 2018 [57]	Not reported	Bega Garnbirringu Health Service	Australia	Western Australia
Maari Ma Health Aboriginal Corporation mobile clinic	Australian Mobile Health Clinics Association 2015 [58] Parliament of Australia 2014 [59]	2014	Maari Ma Health Aboriginal Corporation	Australia	New South Wales
University of Queensland Indigenous Health Mobile Training Unit/Medical Outreach Boomerang van (MOB van)	Australian Mobile Health Clinics Association 2015 [58] University of Queensland 2013 [60] Carbal Medical Service 2020 [61] Carbal Medical Services 2014 [62]	2013	University of Queensland, Health Workforce Australia and Carbal Health Service	Australia	Queensland
Moorditj Djena mobile podiatry clinic	Ballestas et al. 2014 [63]	2011	Derbarl Yerrigan Health Service and North and South Metropolitan Health Services	Australia	Western Australia
Western Desert Kidney Health mobile bus	Bestel 2010 [64] Sinclair et al. 2016 [65] Jeffries-Stokes 2017 [66]	2010	University of Western Australia	Australia	Western Australia
Tulku Wan Wininn mobile clinic	Budja Budja Aboriginal Cooperative 2019 [40]	2019	Budja Budja Aboriginal Cooperative	Australia	Victoria
Queensland Aboriginal and Islander Health Council (QAIHC) mobile health clinic	Burgess & Buchannan 2013 [67]	2013	QAIHC	Australia	Queensland
Goondir Health Services Mobile Medical Clinic (MMC)	Goondir Health Services 2020 [68] Goondir Health Services 2019 [69]	2010	Goondir Health Services	Australia	Queensland
Earbus mobile health clinics	Ear bus 2020 [70] Ear bus 2018 [71]	2014	Earbus foundation of Western Australia	Australia	Western Australia
Chevron-Pilbara Ear Health Program	Telethon Speech & Hearing 2020 [72] Higginbotham & Shur 2012 [73] Krishnaswamy, Monley & Kishida 2015 [74] Telethon Speech & Hearing 2019 [75]	2011	Telethon Speech & Hearing	Australia	Western Australia
Pi:Lu Bus	Evins 2018 [76]	2018	Riverland Aboriginal Health Service	Australia	South Australia
Murchison Outreach Services mobile clinic	Geraldton Regional Aboriginal Medical Service 2020 [77]	Not reported	Geraldton Regional Aboriginal Medical Service	Australia	Western Australia
Nhulundu Health Service Mobile Clinic	Nhulundu Health Service 2016 [78]	Not reported	Nhulundu Health Service	Australia	Queensland
Screening for Limb, I-eye, Cardiovascular, and Kidney complications of diabetes (SLICK vans)	Jin 2014 [79] Oster et al. 2009 [80] Oster et al. 2010a [41] Virani et al. 2006 [81]	2001–2010	University of Alberta, First Nations and Health Canada	Canada	Alberta
Mobile Diabetes Screening Initiative (MDSi)	Ralph-Campbell et al. 2009 [82] Oster et al. 2010b [83] Ralph-Campbell et al. 2011 [84] Toth 2014 [85]	2003	Alberta Health and Wellness, Northern Regional Health Authorities and University of Alberta	Canada	
Seabird Island Mobile Diabetes Telemedicine	Jin 2014 [79]	2009	Seabird Island Band	Canada	British Columbia
Manitoba Diabetes Integration Project (DIP)	Jin 2014 [79]	2008	Diabetes Integration Project, Inc.	Canada	Manitoba
Mobile Diabetes Telemedicine Clinic	First Nations Health Authority 2019 [86] Dawson et al. 2009 [87] Jin 2014 [79] Carrier Sekani Family Services 2015 [88]	2002	Carrier Sekani Family Services	Canada	British Columbia
Great Plains Mobile Mammography Screening	Roubidoux et al. 2018 [89] Roen et al. 2013 [90] Rural Health Information Hub 2019 [91] Indian Health Service 2020 [92]	2006–2018	Great Plains Area Indian Health Service	United States	North and South Dakota, Iowa and Nebraska
Tuba City Regional Health Care Corporation Mobile Health Program	Mobile Healthcare Association 2020 [93] Bylander 2017 [94] Tuba City Regional Health Care Corporation 2019 [95]	Not reported	Tuba City Regional Health Care Corporation	United States	Arizona
Winslow Indian Health Care Centre Medical Mobile Vehicle	Mobile Healthcare Association 2020 [93] Winslow Indian Health Care Centre 2020 [96]	2019	Winslow Indian Health Care Center	United States	Arizona
Bay Clinic Mobile Health Unit	Mobile Health Map 2020 [31] Bay Clinic 2020 [97]	Not reported	Bay Clinic	United States	East Hawaii
Mniwiconi clinic and farm Mobile Clinic	Mobile Health Map 2020 [31] Mniwiconi clinic and farm 2019 [98]	Not reported	Mniwiconi clinic and farm	United States	North Dakota
Wisconsin Ho-Chunk Nation mobile clinic	Children’s Health Fund 2012 [99] Mobile Healthcare Association 2020 [93]	2012	Ho-Chunk Nation Department of Health and Children’s Fund	United States	Wisconsin

**Fig. 2:**
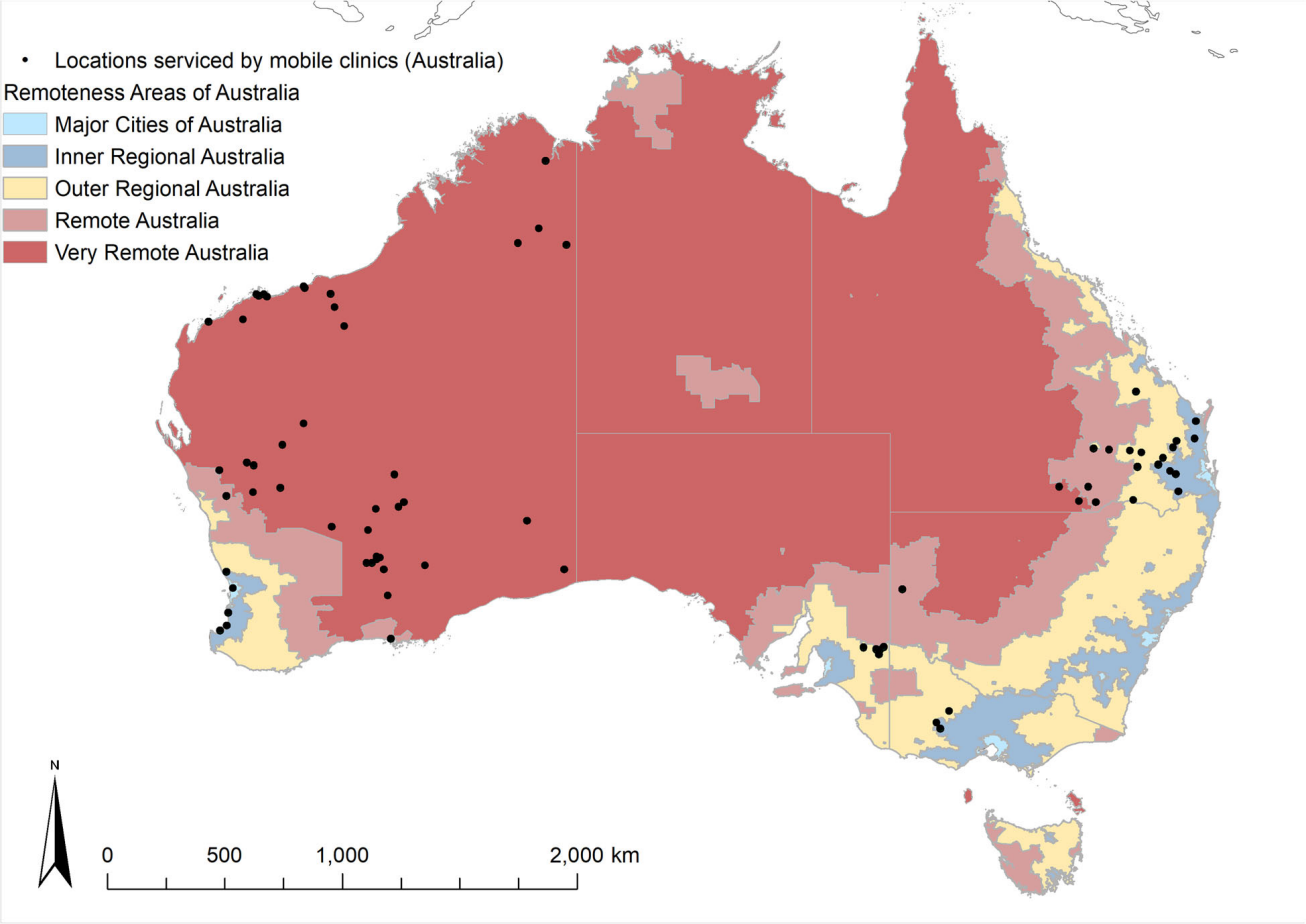
Location of mobile clinics implemented for Indigenous populations in Australia

In Australia, the majority of locations serviced by mobile clinics were located in Very Remote Australia (*n* = 44; Table 3; Fig. 2). This was compared to Inner and Outer Regional Australia, which both had a similar amount of locations represented (*n* = 15 and *n* = 17 respectively). The remoteness classification with the least amount of locations was Major Cities of Australia (*n* = 2).

**Table 3:** Summary of mobile clinics in Australia, Canada and the United States stratified by measure of remoteness or population size

**Australia (Remoteness Structure)**	**Frequency of locations serviced by mobile clinics (%)**
Major Cities of Australia	2 (2.3)
Inner Regional Australia	15 (17.2)
Outer Regional Australia	17 (19.5)
Remote Australia	9 (10.4)
Very Remote Australia	44 (50.6)
Total	87 (100.0)
**Canada (Population Centre and Rural Area Classification 2016)**	
Large Urban (> 100,000)	3 (1.9)
Medium (30,000-99,999)	6 (3.7)
Small (1000–29,999)	11 (6.8)
Outside (< 1000)	142 (87.7)
Total	162 (100.0)
**United States (Urban areas)**	
Urbanised Area (> 50,000)	1 (2.8)
Urbanised Cluster (2500-49,999)	11 (30.6)
Outside classification (< 2499)	24 (66.7)
Total	36 (100.0)

**Fig. 3:**
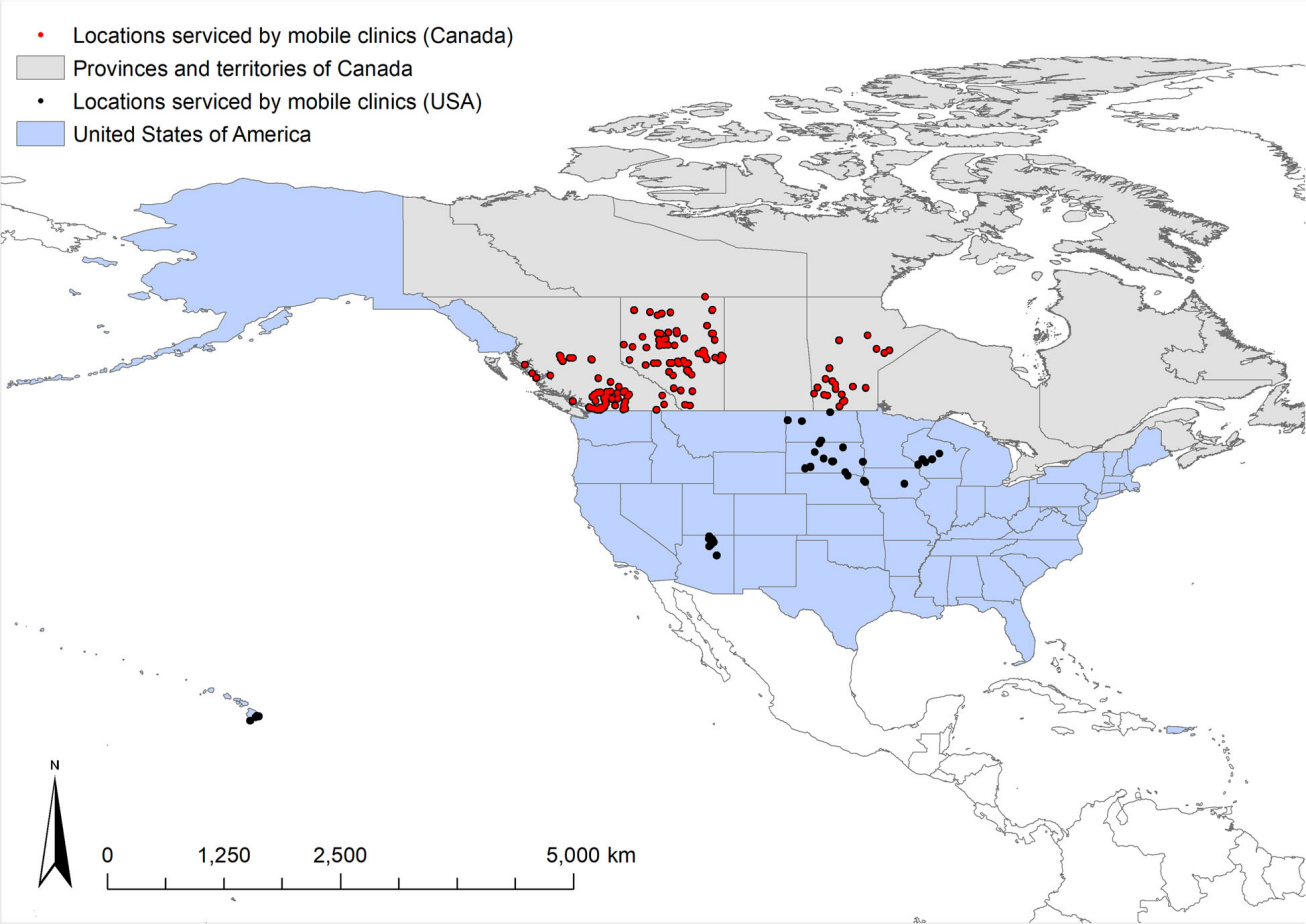
Location of mobile clinics implemented for Indigenous populations in Canada and the United States

In Canada, most locations serviced by a mobile clinic were outside the formal classification of population centres (*n* = 142; Table 3; Fig. 3). There was a declining presence of mobile clinics with the increasing size of population centres. This was similar to the United States where two thirds of mobile clinic activity was in areas classified as being outside Urbanised Areas or Urbanised Clusters (*n* = 24, Table 3; Fig. 3). Locations with a mobile clinic presence were more numerous in Urbanised Clusters (*n* = 11) compared to Urbanised Areas (*n* = 1).

### Finding 2: primary health mobile clinic models for Indigenous populations

Of the mobile clinics included in the search (*n* = 25), the types of primary health care services and targeted populations varied (Table 3). These included delivering a broad range of general primary health care services (*n* = 13), providing disease specific services (e.g. diabetes management, screening and education *n* = 6, renal disease and other chronic disease screening n = 1, breast cancer screening *n* = 1, ear disease screening *n* = 3) and opportunistic health services and health promotion (*n* = 1) to Indigenous populations.

Most of the mobile clinics were implemented for Indigenous populations across the lifespan (*n* = 15), with fewer implemented for a specific age, gender group or population with chronic disease (infants, children or young people aged less than 18 years *n* = 4, people with diabetes n = 4, women *n* = 1, adults *n* = 1). There was evidence of Indigenous organisations governing and/or implementing 14 of the 25 mobile clinics (56%), with the remainder implemented in partnership with a non-Indigenous organisation or institution (*n* = 10). No information was provided about the involvement of Indigenous people in the implementation of one mobile clinic [67].

Information about the funding source(s) was retrieved for 19 of the 25 (76%) mobile clinics. Various sources were used to fund the mobile clinics which included governments, health organisations, commercial entities, universities and philanthropic organisations or foundations.

### Finding 3: evidence of evaluated mobile clinics for Indigenous populations

Of the 25 included mobile clinics, 13 (52%) had evidence of some form of evaluation (Table 4). Of these 13 mobile clinics, most of the evaluation findings were disseminated in the non peer-reviewed literature or grey literature (*n* = 7 mobile clinics), with fewer evaluation findings disseminated in the peer-reviewed literature (*n* = 6 mobile clinics).

Of the evaluated mobile clinics, various approaches to undertaking an evaluation were used. Some evaluations produced multiple citations for a single mobile clinic (Table 4). Most of the evaluations used quantitative methods of evaluation (*n* = 11) including descriptive statistics (e.g. of clinical indicators, patient demographics, service data), surveys and longitudinal data. One of these included a cost-effectiveness analysis [52]. Two evaluations used a mixed methods approach consisting of both quantitative and qualitative methods of evaluation. Of the two mobile clinics evaluated using mixed methods (e.g. including qualitative methods of data collection such as interviews and focus group sessions), one evaluation did not provide qualitative data [63], whereas the other provided rich qualitative findings with evidence of engaging with the perspectives and voices of Indigenous people [65, 66]. Evaluations were heterogeneous in terms of evaluation methods and outcomes, making it difficult to compare findings. However, the participant sample included in evaluations was those receiving the services of the respective mobile clinic with a client or patient record (Table 4).

**Table 4:** Included primary health care mobile clinic models for Indigenous populations

MOBILE PRIMARY HEALTH CARE MODEL							
Mobile health clinic name	Target population	Services providedwd	Indigenous community involvement	Evaluation methods	Participant sample	Evaluation Outcomes	Mobile clinic funding source
Health E Screen 4 Kids	Aboriginal and Torres Strait Islander children (aged > 18 years)	Screening, surveillance, primary care health checks and ENT surgery (e.g. taking out adenoids, putting in grommets)	In partnership	(1) Feasibility study [51] (2) Cost-effectiveness analysis [52] (3) Pre and post-intervention analysis of hospital ENT service utilization [53] (4) Retrospective review of service activity from 2009 to 2011 [55] (5) Retrospective review of service activity from 2009 to 2014 [54]	(1) Aboriginal and Torres Strait Islander children aged between 0 and 16 years receiving service between February and July 2009 (*n* = 743) (2) Annual costs of mobile van including services delivered, staff costs, maintenance costs and fixed costs (3) ENT outpatient appointments at Royal Children’s Hospital (2006–2008 *n* = 329) and (2009–2011 *n* = 105) (4) Children registered with the service (*n* = 1053) (5) Children registered with the service (*n* = 3105)	(1) 41% of children failed one or more components of ear-screening assessment, 12% had signs of hearing impairment and 15% failed vision-screening assessment with 157 referrals to ENT specialists for review (2) Estimated cost for mobile van was higher than control (Deadly Ears Program), however generated high QALYs (15.94 v. 15.90) than control. Found to be a cost-effective strategy (3) Increase in routine assessment of children, increase in ENT surgical procedures locally and reduced need for families to travel to tertiary centers (4) High screening rates of children as a result of service with 2111 screening assessments undertaken and reduced wait times for ENT specialist review (5) Since service commenced, the number of screening assessments completed per year has increased (2009 *n* = 752, compared to 2014 *n* = 1454), increase in patients and decrease in proportion of children failing screening assessments and being referred to ENT.	University of Queensland, Centre for Online Health and Royal Children’s Hospital Foundation
Bega Garnbirringu Health Service mobile clinic	All Aboriginal and Torres Strait Islander people	Primary care services delivered by GP, RN and AHWs such as wound care, health screenings (including sexual health), chronic disease management, pathology services (including Point of Care (PoC) testing), health education, annual Aboriginal and Torres Strait Islander health checks and visiting specialist services.	Implemented and delivered by an Aboriginal Community-Controlled Health Organisation	None to report	None to report	None to report	Not reported
Maari Ma Health Aboriginal Corporation mobile clinic	All Aboriginal and Torres Strait Islander people	Opportunistic health service delivery at community and sports events including health promotion and influenza vaccination	Implemented and delivered by an Aboriginal Community-Controlled Health Organisation	None to report	None to report	None to report	Australian Commonwealth Government
University of Queensland Indigenous Health Mobile Training Unit (MOB van)	All Aboriginal and Torres Strait Islander people	Primary health care including GP assessments, opportunistic health checks and school health checks	Implemented and delivered in partnership with an Aboriginal Community-Controlled Health Organisation	Descriptive statistics for 2014 annual report [62]	Clients of service	Multiple outcomes including 50% increase in the number of active clients, triple the number of GPs employed in 2014 compared to 2013, an increase in the number of completed health checks by 29% and funding secured for a new clinic in Warwick.	Queensland Health, Health Workforce Australia
Moorditj Djena mobile podiatry clinic	All Aboriginal and Torres Strait Islander people	Diabetes self-management and education including podiatric assessment.	Implemented and delivered by an Aboriginal Community -Controlled Health Organisation	Mixed methods including focus groups, interviews, review of program documents and descriptive analysis of clinical and administrative data [63].	Clients of service (*n* = 702)	Multiple outcomes including 3500 occasions of service in first 2.5 years and identified that outreach capacity is a strength. Multiple challenges including planning and coordination of outreach clinics, recruitment of staff and staff turnover, van procurement, launch and ongoing promotion of clinical service, ordering of equipment and logistical organisation, development of a database for electronic record keeping and negotiating fees to minimize costs to clients.	National Partnership Agreement for ‘Closing the Gap’ in Indigenous Health Outcomes
Western Desert Kidney Health mobile bus	All Aboriginal and Torres Strait Islander people	Early detection of disease, chronic disease management and health promotion	Implemented and delivered in partnership with Aboriginal organisations	(1) Qualitative interviews [65] (2) Community based participatory research project with annual cross sectional surveys over 3 years [66]	(1) Aboriginal people living in remote communities receiving service (n = 26) (2) Aboriginal people from 10 locations	(1) Found to be highly acceptable and effective means of disseminating the importance of prevention, early detection and management of diabetes and kidney disease (2) Multiple outcomes including high participation rate of Aboriginal people (79%), higher than predicated rates of diabetes, hypertension, hematuria and ACR and Aboriginal women found to be the highest risk group	BHP Billiton Nickel West, University Western Australia, University of Notre Dame, Bega Garnbirringu Health Services, Goldfields Esperance GP Network and Wongutha Bimi Aboriginal Corporation
Budja Budja Aboriginal Cooperative	All Aboriginal and Torres Strait Islander people	Primary health care services including audiology, optometry, general health checks and health promotion and education	Implemented and delivered	None to report	None to report	None to report	Deakin University School of Medicine, Department of Prime Minister and Cabinet (Indigenous Affairs) and Budja Budja Aboriginal Coopertaive
Queensland Aboriginal and Islander Health Council (QAIHC) mobile health clinic	Aboriginal and Torres Strait Islander people	Primary health care services	Not reported	None to report	None to report	None to report	Queensland Gas Company (QGC)
Goondir Health Services Mobile Medical Clinic	Aboriginal and Torres Strait Islander people, including school-aged children	Primary health care including disease prevention and chronic disease management, men’s and women’s health and health checks in schools	Delivered by an Aboriginal Community Controlled Health Organisation	Descriptive statistics of services delivered [69]	Clinic data	Multiple outcomes including 187% increase in number of patients over 4 years.	Queensland Health, Broncos and Goondir Health Service
Earbus mobile health clinics	Aboriginal and Torres Strait Islander children and young people	Primary and secondary services including ear screening, surveillance and treatment by GPs, audiologist and ENTs	Partners with Aboriginal Community-Controlled Health Service to deliver health services.	Regional descriptive statistics of services delivered [71]	Patient records	Multiple outcomes reporting on disease prevalence and screening rates in patient cohort stratified by geographical region	Earbus foundation of WA (charity) receiving multiple sources of funding (e.g. Neilson Foundation, ALCOA, MZI Resources and Ian Potter Foundation)
Chevron-Pilbara Ear Health Program	Aboriginal and Torres Strait Islander school-aged children	Primary and secondary services including: ear health checks, hearing screening, Nurse Practitioner consultations and appointments with Ear Nose and Throat Specialists.	Partner with Aboriginal communities, Elders, schools and other health services to deliver health services.	(1) Descriptive statistics of services delivered [73] (2) Descriptive statistics of attendance rates [74] (3) Descriptive statistics in annual report [75]	(1) Patient and clinical data (2) Clinical data 2014–2015 (3) Clinical data 2011–2019	(1) Multiple outcomes including number of schools accessed and outcomes of hearing tests (pass, review, refer) (2) Increased attendance rates (40% pre July 2014 to 85.1% Jan-June 2015) (3) Multiple outcomes including 10,137 ear health screenings for 4881 people.	Partners, Benefactors & Supporters; Channel 7 Telethon Trust, Chevron, Western Australian Government, Lottery West, The Hearing Research & Support Foundation, The Crommelin Family, Bill 7 Rhonda Wyllie Foundation, Jack Bendat, Tony Fini Foundation, Stan Perron Charitable Trust, Frank Tomasi Family Trust, Toybox International, LD Total
Pi:Lu Bus	All Aboriginal and Torres Strait Islander people	Primary health services including education	Aboriginal health service delivered	None to report	None to report	None to report	Bus provided by Transport South Australia
Murchison Outreach Services	All Aboriginal and Torres Strait Islander people	Primary care services including: general medical care, chronic disease and health promotion	Operated and delivered by an Aboriginal Community Controlled Health Organisation	None to report	None to report	None to report	Not reported
Nhulundu Health Service Mobile Clinic	All Aboriginal and Torres Strait Islander people	Outreach medical services delivered by a GP, nurse and health worker	Operated and delivered by an Aboriginal medical service	None to report	None to report	None to report	Not reported
Screening for Limb, I-eye, Cardiovascular, and Kidney complications of diabetes (SLICK vans)	Alberta First Nations with diabetes	Diabetes screening, education and counselling service with point-of-care laboratory equipment and a retinal camera	Delivered in partnership with First Nations people	(1) Descriptive analysis of patient cohort [80] (2) Descriptive longitudinal analysis of clinical indicators [41] (3) Descriptive quantitative analysis of key evaluation indicators [79] (4) Preliminary evaluation [81]	(1) Participants who completed screening and survey (n = 743) (2) Patients screened with diabetes 2001–2007 (*n* = 2102) (3) Patient and clinic data between 2001 and 2007 (4) First Nations people with known diabetes 2001 to 2003	(1) Various clinical indicators, service utilization and health literacy. (2) Significant improvements in BMI, blood pressure, total cholesterol and HbA1c were identified (*p* < 0.01) in returning patients (3) Multiple outcomes including clinic visits (*n* = 830), annual costs avoided by patients and changes in clinical indicators (e.g. mass (kg), BMI, HbA1c, BP, MAP, LDL and total cholesterol) (4) Screened *n* = 1151 clients, modest improvements in program outcomes at 6 to 12 months	Canadian Health Infostructure Partnership Program (CHIPP), Health Canada and Aboriginal Diabetes Initiative
Mobile Diabetes Screening Initiative (MDSi)	Metis adults (aged 18 years and over) and other remote Indigenous communities	Diabetes screening service	Implemented in partnership with Metis communities	(1) Descriptive cross-sectional quantitative study with multiple measures (e.g. body mass index (BMI), waist circumference, blood pressure, blood glucose, blood lipids and HbA1c) [82] (2) Telephone survey [83] (3) Longitudinal analysis [84]	(1) Patients screened with fasting glucose without a known history of diabetes (*n* = 266) between 2003 and 2007 (2) Adult patients (*n* = 175) between 2003 and 2008 (3) Clinical data from 2003 to 2009	(1) Prevalence of undiagnosed diabetes was 5.3% and pre-diabetes was 20.3% (CDA criteria) and 51.9% (ADA criteria) (2) 51% of participants indicated GP follow up after screening, with 66% of those who had been told they had probable diabetes, visiting a physician. (3) For returning adults with diabetes, significant improvements (*p* < 0.05) were observed in BMI, blood pressure, total cholesterol and HbA1c.	Alberta Health and Wellness Program
Seabird Island Mobile Diabetes Telemedicine	People with diabetes residing in 70 First Nation reserve communities in southern mainland BC	Eye exam, PoC laboratory tests, nurse assessment, diabetes management and education	Directed by members from tribal councils	Descriptive quantitative analysis of key evaluation indicators [79]	Patient records with diabetes (*n* = 1160) 2010–2013	Multiple outcomes including patient mean avoided cost ($260,027 per year) and mean difference in clinical indicators of diabetes (although not statistically significant): body mass − 0.5 kg, HbA1c −0.08%, systolic blood pressure 1.1 mmHg, diastolic blood pressure − 0.5 mmHg, Mean Arterial Pressure (MAP) 0.1 mmHg, Low Density Lipids (LDL) -0.13 mmol/L	Health Canada, Aboriginal Diabetes Initiative and British Columbia agencies including Fraser Health Authority and First Nations Health Authority.
Manitoba Diabetes Integration Project (DIP)	People with diabetes residing in 19 First Nation reserves in Manitoba	PoC laboratory tests, nurse assessment and diabetes management and education advice	Directed by members from tribal councils	Descriptive quantitative analysis of key evaluation indicators [79]	Patient records with diabetes (*n* = 2790) between 2008 and 2013	Multiple outcomes including patient mean avoided cost ($272,289 per year) and change in mean difference of clinical indicators of diabetes: mass − 0.4 kg, HbA1c − 0.09%, systolic blood pressure − 1.6 mmHg, diastolic blood pressure − 1.0 mmHg, MAP −1.1 mmHg, LDL 0.09 mmol/L	Health Canada and Aboriginal Diabetes Initiative
Mobile Diabetes Telemedicine Clinic (MDTC)	People with diabetes residing in 59 First Nations communities in Northern British Columbia	Diabetes screening and management including eye exam, point of care (PoC) testing, nursing and dietitian assessments and education	Delivered by First Nations health service	Longitudinal cohort data analysis [79, 87]	Patient records from 2003 to 2009	Modest improvements in some clinical outcomes (e.g. mean decline in body mass of 1.6 kg, mean decline in LDL was 0.3 mmol/L, mean absolute decline in A1c was 0.4%)	Health Canada and Aboriginal Diabetes Initiative
Great Plains Mobile Mammography Screening	Native American and Alaskan women	Mammography screening and referrals to tertiary centers	Delivered by Indian health services	(1) Retrospective analysis of clinic records [89] (2) Retrospective analysis of clinical records [90]	(1) Native Indian and Alaskan Native patient records 2007–2009 (*n* = 2640) (2) Complete patient records from 2007 to 2009 (*n* = 1771)	(1) Incomplete patient reports were more frequent in mobile mammography than the fixed site (21.9% v. 15.2%) (2) Adherence to screening guidelines found in 39.86% of patients	Not reported
Tuba City Regional Health Care Corporation Mobile Health Program	Native Indian people from Navajo, Hopi and San Juan Southern Paiute tribes	Primary Healthcare including immunizations and dental exams	Delivered by an Indian Tribal Organisation	None to report	None to report	None to report	Health Resources and Services Administration (Grant)
Winslow Indian Health Care Centre Medical Mobile Vehicle	Native Indian people	Primary care, dental, pharmacy, public health nursing, physical therapy, and some specialty services.	Delivered by Indian health service	None to report	None to report	None to report	Not reported
Bay Clinic Mobile Health Unit	East Hawai’i residents	Primary health care including preventative care, treatment, urgent care, immunization and vaccines, chronic disease management and dental services	Delivered by an East Hawai’I community health service	None to report	None to report	None to report	The Harry & Jeanette Weinberg Foundation, Inc., Hearst Foundations, Atherton Family Foundation, HDS Foundation, USDA/Rural Development, County of Hawai’i, McInerny Foundation, Ouida & Doc Hill Foundation, The Shippers Wharf Committee Trust
Mniwiconi clinic and farm Mobile Clinic	Indian tribal members	Health care	Indian delivered	None to report	None to report	None to report	Not reported
Wisconsin Ho-Chunk Nation mobile clinic	Indian babies, children and young people	Primary healthcare including acute care, laboratory services, vision and hearing screening, immunisations and other preventative care, education (e.g. asthma management, obesity prevention)	Delivered in partnership with an Indian Department of Health	None to report	None to report	None to report	Idol Gives Back Foundation (philanthropic)

## Discussion

To our knowledge, this is the first systematic scoping review examining primary health care mobile clinics implemented for Indigenous populations in Australia, Canada, New Zealand, and the United States. This review locates evidence of mobile clinics that have been implemented specifically for Indigenous populations (with the exception of New Zealand), and highlights the potential for mobile clinics to improve the accessibility of primary health care services. These findings are a valuable contribution to the growing body of international literature around the use of mobile clinics [28, 29, 32, 33, 36, 38]. Before discussing the implications of these findings, it is important to reiterate that Indigenous populations are diverse, have different languages, cultural identities, customs, lore and spiritual beliefs [16]. However, Indigenous populations in Australia, Canada, New Zealand, and the United States share the experience of colonization and require culturally safe health care embedded in the principles of self-determination [7, 16, 17, 46].

Likewise, there are key differences between the health care systems of Australia, Canada, New Zealand, and the United States, which may account for variations in the implementation of mobile clinics specifically for Indigenous populations. Australia, Canada, and New Zealand have universal access to health care for all populations [100–102] which differs from the partially-funded health care system in the United States [103]. There are also complexities around the policies of each modern state regarding the funding of Indigenous-governed health services and programs [104]. In the United States, funding is allocated through the Indian Health Service (IHS), with a key criticism being the failure to provide sufficient resources to meet the health care needs (particularly primary health care needs) of a growing Native American, Native Alaskan, and Native Hawaiian population [17, 105]. In Australia and Canada, Indigenous health organisations (e.g. ACCHOs in Australia and on-reserve First Nations health services in Canada) receive some funding from governments to provide primary health care services to Indigenous populations, yet inequities exist in the distribution of funding (e.g. lack of funding for Métis People) and power imbalances between government and Indigenous health-organisations [17, 27, 104]. The funding structure in New Zealand differs again, with a more integrated approach of health service delivery through mainstream health services or private agencies and greater participation of Māori People in the process of informing the policy of District Health Boards (DHB) [17, 106]. The need to reform health care systems for the provision of equitable and culturally safe health care for Indigenous populations, has been widely discussed in the peer-reviewed literature [27, 104, 105].

There are also variations as to how population density is described in Australia, Canada, and the United States, which also has implications for interpreting the findings of this review (see Table 3). Australia’s Remoteness Structure [47] has a complete coverage of the continent, whereas Canada and the United States classify their urban areas by population size [48, 49]. Although there are other geographical methods for classifying population density (e.g. in Australia, Modified Monash Model [107]), this review has included classification methods used by decision-makers in each country at the time of analysis. For example, the Australian Government’s Rural Health Multidisciplinary Training (RHMT) Program [108] utilizes the Remoteness Structure [47] to guide investment to improve the recruitment and retention of health professionals in rural and remote Australia. Likewise, the Population Centre and Rural Area Classification 2016 (Canada) [48] and Urban status (United States) [49] are both based on the most recent census for each respective country and are used in government decision-making. Acknowledging these variations, this review identifies a pattern of increasing presence of mobile clinics in areas with lower population densities (see Table 3). Geographical gaps in service provision are evident (Figs. 2 and 3), indicating that the implementation of mobile clinics for Indigenous populations is not widespread.

There are also variations in the models of primary health care mobile clinics implemented for Indigenous populations. Most of the mobile clinics retrieved by this review targeted Indigenous populations across the lifespan, indicating a holistic family-centered model of primary health care, which is a preferred characteristic of Indigenous primary health care services [109]. Some mobile clinics targeted specific chronic diseases prevalent in Indigenous populations (e.g. diabetes) [110] and prevention of chronic disease for specific populations (e.g. otitis media in Aboriginal and Torres Strait Islander children) [111]. Although there was some evidence of Indigenous organisational governance or involvement in the implementation of most mobile clinics, it was difficult to ascertain the degree of Indigenous community ownership. This is a key issue which has been discussed in another review examining chronic disease programs implemented for Aboriginal and Torres Strait Islander populations [112], and in the international literature examining health services and programs for Indigenous populations [27, 113, 114]. Indigenous community ownership of mobile clinics is imperative to ensuring culture, self-determination, and community participation are embedded in the delivery of primary health care services [109].

A paucity of published and publicly available evaluations of primary health care mobile clinics implemented specifically for Indigenous populations is also highlighted. This is despite a growing body of literature evaluating mobile clinics implemented for general populations and those at-risk for developing chronic disease, particularly in the United States [28, 31–33, 36, 39]. Although there is heterogeneity in the approaches used to evaluate mobile clinics implemented for Indigenous populations, there is some evidence that supports the potential for mobile clinics to increase attendance rates to services [54, 62, 69, 72] and improve clinical indicators (e.g. BMI, HbA1C) of targeted chronic diseases (e.g. diabetes) in Indigenous people accessing mobile clinic services [41, 79]. However, evaluation methods have relied heavily on the analysis of patient records and service data (see Table 4). The perspectives and insights of Indigenous people accessing mobile clinic services is largely absent. Findings support the need for high quality evaluations of Indigenous health programs which integrate qualitative evidence regarding the views and perspectives of Indigenous people [115]. An absence of qualitative data around the effectiveness of mobile clinics makes it difficult to know whether mobile clinics have potential to improve the cultural accessibility of primary health care services for Indigenous populations. This is a gap in existing knowledge which requires further research.

It is also difficult to examine how sustainable primary health care mobile clinics are when implemented for Indigenous populations. It is noted that the five diabetes mobile clinics retrieved from Canada were funded under the Aboriginal Diabetes Initiative (ADI), yet it is difficult to identify from the available literature as to whether all of these mobile clinics have been sustained over time under the original funding arrangement [79]. This highlights a key issue mediating the sustainability of mobile clinics in general, being the reliance on multiple funding sources (e.g. government and philanthropic) and/or short funding cycles [33]. There is also limited cost-effectiveness data around the use of mobile clinics for Indigenous populations [52]. Future research should include economic evaluations, coupled with an evaluation of the effectiveness and cultural acceptability of mobile clinics for Indigenous populations. This is imperative to informing the allocation of resources by decision-makers (e.g. governments and Indigenous-health organisations) to mobile clinics.

### Limitations

Every effort has been made to search academic databases and grey literature sources for primary health care mobile clinics that have been implemented for Indigenous populations in Australia, Canada, New Zealand, and the United States. In Australia, it is known that a significant proportion of health research involving Aboriginal and Torres Strait Islander populations is published in the grey literature [116]. A thorough search of grey literature information sources across key websites has been undertaken through the independent searching of two researchers and follow up of organisations, authors and researchers for additional information. Therefore, a limitation of this review is the manual processes required to undertake this search and the acknowledgement that there is the potential for some mobile clinics to be missed due to this.

## Conclusions

This review identifies geographical gaps and a paucity of evidence around the implementation of primary health care mobile clinics for Indigenous populations in Australia, Canada, New Zealand and the United States. The findings support the need to undertake rigorous mixed methods evaluations of primary health care mobile clinics implemented specifically for Indigenous populations. Through the involvement of Indigenous people in the evaluation process, greater insights will be obtained as to the potential for mobile clinics to improve access to culturally safe and holistic primary health care services. It is important for organisations implementing primary health mobile clinics for Indigenous populations, to share their experiences by making evaluations publicly available, ideally through the peer-reviewed literature. This is essential in developing evidence around innovative models of health care that have the potential to improve health outcomes for Indigenous people globally. Dissemination of evaluation evidence concerning mobile clinics will also be invaluable to decision-makers, including Indigenous health organisations, who are considering allocating resources to a primary health care mobile clinic.

## Supplementary Information

**Additional file 1.**

**Additional file 2.**

**Additional file 3.**

**Additional file 4.**

## Data Availability

Geographical locations of mobile clinics are publicly available.

## Abbreviations

ACCHO: Aboriginal Community Controlled Health Organisation
JBI: Joanna Briggs Institute
PCC: Population, Concept, Context
PRISMA - ScR: Preferred Reporting Items for Systematic Scoping Reviews
