# Supplementary material for: Mobile primary health care clinics for Indigenous populations in Australia, Canada, New Zealand and the United States: a systematic scoping review
Source: Int J Equity Health. 2020 Nov 9;19:201. doi: 10.1186/s12939-020-01306-0 (PMC7652411; doi:10.1186/s12939-020-01306-0)
Supplement: Supplementary file 2 — Additional file 2. [file 12939_2020_1306_MOESM2_ESM.docx]

**Additional file 2: Electronic search results and terms**

| Ovid MEDLINE | 528 |
| --- | --- |
| CINAHL (EBSCOhost) | 216 |
| Embase (Elsevier) | 788 |
| SocIndex (EBSCOhost) | 16 |
| Cochrane Database of Systematic Reviews | 11 |
| INFORMIT | 113 |
| Total | 1672 |
| Pre 2006 | 60 |
| Duplicate papers | 299 |
| Total left to screen | 1,313 |

Database searches were conducted on the 27 August 2019 and 2 September 2019. Gray literature searches were conducted between 19 August 2019 and 17 September 2019.

Database(s): **Ovid MEDLINE(R) Epub Ahead of Print, In-Process & Other Non-Indexed Citations, Ovid MEDLINE(R) Daily, Ovid MEDLINE and Versions(R)**

Search strategy:

| **#** | **Query** |
| --- | --- |
| 1 | (Australia* or Tasmania* or Victoria* or New South Wales* or Queensland* or Northern Territory* or Western Australia* or South Australia*).tw,kf. or exp Australia/ |
| 2 | (Aborig* or First People* or First Nation*1 or Indigenous or Native* or Islander* or "Torres Strait Islander" or Tribe* or Tribal).tw,kf. |
| 3 | Oceanic Ancestry Group/ or Health Services, Indigenous/ |
| 4 | 2 or 3 |
| 5 | 1 and 4 |
| 6 | (New Zealand* or Northland or Auckland or Waikato or Bay of Plenty or Gisborne or Hawkes Bay or Taranaki or Whanganui or Manawatu or Wellington or Marlborough or Nelson or Tasman or West Coast or Canterbury or Otago or Southland).tw,kf. or exp New Zealand/ |
| 7 | (Aborig* or First People* or First Nation*1 or Indigenous or Native* or Maori or Iwi or Hapu or Tribe* or Tribal).tw,kf. |
| 8 | Oceanic Ancestry Group/ or Health services, Indigenous/ |
| 9 | 7 or 8 |
| 10 | 6 and 9 |
| 11 | (North America* or United States or USA or American).tw,kf. or exp United States/ |
| 12 | Canada*.tw,kf. or exp Canada/ |
| 13 | 11 or 12 |
| 14 | (Native or Indigenous or First Nation*1 or First People* or Tribe* or Tribal or Indian*1 or Nation*1 or Inuit* or Aborig*).tw,kf. |
| 15 | Indians, North American/ or Alaska Natives/ or Inuits/ or Health services, Indigenous/ |
| 16 | 14 or 15 |
| 17 | 13 and 16 |
| 18 | 5 or 10 or 17 |
| 19 | mobile health unit/ |
| 20 | (mobile outreach or mobile outreach clinic*1 or mobile health or mobile clinic*1 or mobile health clinic*1 or mobile health care clinic*1 or mobile primary health care clinic*1 or mobile unit*1 or mobile health unit*1 or mobile health care unit*1 or mobile primary health care unit*1 or mobile facilit*3 or mobile health facilit*3 or mobile health care facilit*3 or mobile primary health care facilit*3).tw,kf. |
| 21 | ((health adj3 van) or (health adj3 bus)).tw,kf. |
| 22 | 19 or 20 or 21 |
| 23 | (ambulatory health service*1 or ambulatory health care service*1 or ambulatory primary health care service*1).tw,kf or Ambulatory care/ or exp Ambulatory care facilities/ |
| 24 | (health service*1 access or health service*1 accessibility or health care service*1 access or health care service*1 accessibility).tw,kf. or exp Delivery of health care/ |
| 25 | exp Health promotion/ or exp Population health/ or exp Primary health care/ |
| 26 | 24 or 25 |
| 27 | (mobile or mobiles or wheel based or travelling or outreach).tw,kf. |
| 28 | 26 and 27 |
| 29 | 22 or 23 or 28 |
| 30 | 18 and 29 |
| 31 | (disaster or disaster relief or disaster response).tw,kf. |
| 32 | (animals not (humans and animals)).sh. |
| 33 | 31 or 32 |
| 34 | 30 not 33 |
| 35 | limit 34 to yr="2006-Current" |

Database(s): **CINAHL (EBSCOhost)**

Search strategy:

| **#** | **Query** |
| --- | --- |
| S1 | TI (Australia* OR Tasmania* OR Victoria* OR New South Wales* OR Queensland* OR “Northern Territory*" OR “Western Australia*” OR “South Australia*”) OR AB ((Australia* OR Tasmania* OR Victoria* OR “New South Wales*” OR Queensland* OR “Northern Territory*” OR “Western Australia*” OR “South Australia*”) |
| S2 | (MH “Australia+”) |
| S3 | S1 OR S2 |
| S4 | TI (Aborig* OR “First People*” OR “First Nation” OR “First Nations” OR Indigenous OR Native* OR Islander* OR "Torres Strait Islander" OR Tribe* OR Tribal) OR AB (Aborig* OR “First People*” OR “First Nation” OR “First Nations” OR Indigenous OR Native* OR Islander* OR "Torres Strait Islander" OR Tribe* OR Tribal) |
| S5 | (MH “Aboriginal Australians”) |
| S6 | (MH “Health Services, Indigenous”) |
| S7 | S4 OR S5 OR S6 |
| S8 | S3 AND S7 |
| S9 | TI (“New Zealand*” OR Northland OR Auckland OR Waikato OR “Bay of Plenty” OR Gisborne OR “Hawkes Bay” OR Taranaki OR Whanganui OR Manawatu OR Wellington OR Marlborough OR Nelson OR Tasman OR “West Coast” OR Canterbury OR Otago OR Southland) OR AB (“New Zealand*” OR Northland OR Auckland OR Waikato OR “Bay of Plenty” OR Gisborne OR “Hawkes Bay” OR Taranaki OR Whanganui OR Manawatu OR Wellington OR Marlborough OR Nelson OR Tasman OR “West Coast” OR Canterbury OR Otago OR Southland) |
| S10 | (MH “New Zealand+”) |
| S11 | S9 OR S10 |
| S12 | TI (Aborig* OR “First People*” OR “First Nation” OR “First Nations” OR Indigenous OR Native* OR Maori OR Iwi OR Hapu OR Tribe* OR Tribal) OR AB (Aborig* OR “First People*” OR “First Nation” OR “First Nations” OR Indigenous OR Native* OR Maori OR Iwi OR Hapu OR Tribe* OR Tribal) |
| S13 | (MH “Maori”) |
| S14 | (MH “Health Services, Indigenous”) |
| S15 | S12 OR S13 OR S14 |
| S16 | S11 AND S15 |
| S17 | TI (“North America*” OR “United States” OR USA OR American) OR AB (“North America*” OR “United States” OR USA OR American) |
| S18 | (MH “United States+”) |
| S19 | TI (Canada*) OR AB (Canada*) |
| S20 | (MH “Canada+”) |
| S21 | S17 OR S18 OR S19 OR S20 |
| S22 | TI (Native OR Indigenous OR “First Nation” OR “First Nations” OR “First People*” OR Tribe* OR Tribal OR Indian* OR Nation OR Nations OR Inuit* OR Aborig*) OR AB (Native OR Indigenous OR “First Nation” OR “First Nations” OR “First People*” OR Tribe* OR Tribal OR Indian* OR Nation OR Nations OR Inuit* OR Aborig*) |
| S23 | (MH “Native Americans”) |
| S24 | (MH “Inuit”) |
| S25 | (MH “Health Services, Indigenous”) |
| S26 | S22 OR S23 OR S24 OR S25 |
| S27 | S21 AND S26 |
| S28 | S8 OR S16 OR S27 |
| S29 | (MH “mobile health units”) |
| S30 | TI (“mobile outreach” OR “mobile outreach clinic” OR “mobile outreach clinics” OR “mobile health” OR “mobile clinic” OR “mobile clinics” OR “mobile health clinic” OR “mobile health clinics” OR “mobile health care clinic” OR “mobile health care clinics” OR “mobile primary health care clinic” OR “mobile health care clinics” OR “mobile health unit*” OR “mobile health care unit*” OR “mobile primary health care unit*” OR “mobile facilit*” OR “mobile health facilit*” OR “mobile health care facilit*” or “mobile primary health care facilit*”) OR AB (“mobile outreach” OR “mobile outreach clinic” OR “mobile outreach clinics” OR “mobile health” OR “mobile clinic” OR “mobile clinics” OR “mobile health clinic” OR “mobile health clinics” OR “mobile health care clinic” OR “mobile health care clinics” OR “mobile primary health care clinic” OR “mobile health care clinics” OR “mobile health unit*” OR “mobile health care unit*” OR “mobile primary health care unit*” OR “mobile facilit*” OR “mobile health facilit*” OR “mobile health care facilit*” or “mobile primary health care facilit*”) |
| S31 | TI (health N3 (van OR bus)) OR AB (health N3 (van OR bus)) |
| S32 | S29 OR S30 OR S31 |
| S33 | TI (ambulatory health service* OR ambulatory health care service* OR ambulatory primary health care service*) OR AB (ambulatory health service* OR ambulatory health care service* OR ambulatory primary health care service*) |
| S34 | (MH “Ambulatory care”) |
| S35 | (MH “Ambulatory care facilities+”) |
| S36 | S33 OR S34 OR S35 |
| S37 | TI (“health service* access” OR “health service* accessibility” OR “health care service* access” OR “health care service* accessibility”) OR AB (“health service* access” OR “health service* accessibility” OR “health care service* access” OR “health care service* accessibility”) |
| S38 | (MH “Health care delivery+”) |
| S39 | (MH “Health promotion+”) |
| S40 | (MH “Population health”) |
| S41 | (MH “Primary health care”) |
| S42 | S37 OR S38 OR S39 OR S40 OR S41 |
| S43 | TI (mobile OR mobiles OR “wheel based” OR travelling OR outreach) OR AB (mobile OR mobiles OR “wheel based” OR travelling OR outreach) |
| S44 | S42 AND S43 |
| S45 | S32 OR S36 OR S44 |
| S46 | S28 AND S45 |
| S47 | TI (disaster OR “disaster relief” OR “disaster response”) OR AB (disaster OR “disaster relief” OR “disaster response”) |
| S48 | S46 NOT S47 |
| S49 | Limiters: Published Date: 20060101-20191231 |

Database(s): **Embase (Elsevier)**

Search strategy:

| **#** | **Query** |
| --- | --- |
| 1 | (Australia* OR Tasmania* OR Victoria* OR "New South Wales*" OR Queensland* OR "Northern Territory*" OR "Western Australia*" OR "South Australia*"):ti,ab,kw |
| 2 | “Australia”/exp |
| 3 | #1 OR #2 |
| 4 | (Aborig* OR "First People*" OR "First Nation” OR “First Nations” OR Indigenous OR Native* OR Islander* OR "Torres Strait Islander" OR Tribe* OR Tribal):ti,ab,kw |
| 5 | “Oceanic Ancestry Group”/exp |
| 6 | “Indigenous Health Care”/de |
| 7 | #4 OR #5 OR #6 |
| 8 | #3 AND #7 |
| 9 | ("New Zealand*" OR Northland OR Auckland OR Waikato OR "Bay of Plenty" OR Gisborne OR "Hawkes Bay" OR Taranaki OR Whanganui OR Manawatu OR Wellington OR Marlborough OR Nelson OR Tasman OR "West Coast" OR Canterbury OR Otago OR Southland):ti,ab,kw |
| 10 | “New Zealand”/exp |
| 11 | #9 OR #10 |
| 12 | (Aborig* OR "First People*" OR "First Nation" OR “First Nations” OR Indigenous OR Native* OR Maori OR Iwi OR Hapu OR Tribe* OR Tribal):ti,ab,kw |
| 13 | “Oceanic Ancestry Group”/exp |
| 14 | “Indigenous Health Care”/de |
| 15 | #12 OR #13 OR #14 |
| 16 | #11 AND #15 |
| 17 | ("North America*" OR "United States" OR USA OR American):ti,ab,kw |
| 18 | “United States”/exp |
| 19 | Canada*:ti,ab,kw |
| 20 | “Canada”/exp |
| 21 | #17 OR #18 OR #19 OR #20 |
| 22 | (Native OR Indigenous OR "First Nation" OR “First Nations” OR "First People*" OR Tribe* OR Tribal OR Indian OR Indians OR Nation OR Nations OR Inuit* OR Aborig*):ti,ab,kw |
| 23 | “American Indian”/de |
| 24 | “Alaska Native”/de |
| 25 | “Inuit”/exp |
| 26 | “Indigenous Health Care”/de |
| 27 | #22 OR #23 OR #24 OR #25 OR #26 |
| 28 | #21 AND #27 |
| 29 | #8 OR #16 OR #28 |
| 30 | “Preventive Health Service”/exp |
| 31 | (“mobile outreach” OR “mobile outreach clinic” OR “mobile outreach clinics” OR “mobile health” OR “mobile clinic” OR “mobile clinics” OR “mobile health clinic” OR “mobile health clinics” OR “mobile health care clinic” OR “mobile health care clinics” OR “mobile primary health care clinic” OR “mobile primary health care clinics” OR “mobile health unit” OR “mobile health units” OR “mobile health care unit” OR “mobile health care units” OR “mobile primary health care unit” OR “mobile primary health care units” OR “mobile facilit*” OR “mobile health facilit*” OR “mobile health care facilit*” or “mobile primary health care facilit*”):ti,ab,kw |
| 32 | (heath NEAR/3 (van OR bus)):ti,ab,kw |
| 33 | #30 OR #31 OR #32 |
| 34 | ("ambulatory health service*" OR "ambulatory health care service*" OR "ambulatory primary health care service*"):ti,ab,kw |
| 35 | “Ambulatory care”/exp |
| 36 | “Outpatient department”/de |
| 37 | #34 OR #35 OR #36 |
| 38 | ("health service* access" OR "health service* accessibility" OR "health care service* access" OR "health care service* accessibility"):ti,ab,kw |
| 39 | “Health care delivery”/exp |
| 40 | “Health promotion”/exp |
| 41 | “Population health”/de |
| 42 | “Primary health care”/exp |
| 43 | #38 OR #39 OR #40 OR #41 OR #42 |
| 44 | (mobile OR mobiles OR "wheel based" OR travelling OR outreach):ti,ab,kw |
| 45 | #43 AND #44 |
| 46 | #33 OR #37 OR #45 |
| 47 | #29 AND #46 |
| 48 | (disaster OR "disaster relief" OR "disaster response"):ti,ab,kw |
| 49 | #47 NOT #48 |
| 50 | #49 NOT ([animals]/lim NOT [humans]/lim) |
| 51 | #50 AND [2006-2019]/py |

# Database(s):SocINDEX (EBSCOhost)

# Search Strategy:

| **#** | **Query** |
| --- | --- |
| S1 | TI (Australia* or Tasmania* or Victoria* or “New South Wales*” or Queensland* or “Northern Territory*” or “Western Australia*” or “South Australia*”) OR AB (Australia* or Tasmania* or Victoria* or “New South Wales*” or Queensland* or “Northern Territory*” or “Western Australia*” or “South Australia*”) OR KW (Australia* or Tasmania* or Victoria* or “New South Wales*” or Queensland* or “Northern Territory*” or “Western Australia*” or “South Australia*”) |
| S2 | TI (Aborig* or “First People*” or “First Nation” or “First Nations” or Indigenous or Native* or Islander* or "Torres Strait Islander" or Tribe* or Tribal) OR AB ((Aborig* or “First People*” or “First Nation” or “First Nations” or Indigenous or Native* or Islander* or "Torres Strait Islander" or Tribe* or Tribal) OR KW (Aborig* or “First People*” or “First Nation” or “First Nations” or Indigenous or Native* or Islander* or "Torres Strait Islander" or Tribe* or Tribal) |
| S3 | DE “Health of Indigenous peoples” |
| S4 | (DE "Indigenous Peoples” OR DE “Indigenous Children” OR DE “Indigenous youth") |
| S5 | S2 OR S3 OR S4 |
| S6 | S1 AND S5 |
| S7 | TI (“New Zealand*” or Northland or Auckland or Waikato or “Bay of Plenty” or Gisborne or “Hawkes Bay” or Taranaki or Whanganui or Manawatu or Wellington or Marlborough or Nelson or Tasman or “West Coast” or Canterbury or Otago or Southland) OR AB (“New Zealand*” or Northland or Auckland or Waikato or “Bay of Plenty” or Gisborne or “Hawkes Bay” or Taranaki or Whanganui or Manawatu or Wellington or Marlborough or Nelson or Tasman or “West Coast” or Canterbury or Otago or Southland) OR KW (“New Zealand*” or Northland or Auckland or Waikato or “Bay of Plenty” or Gisborne or “Hawkes Bay” or Taranaki or Whanganui or Manawatu or Wellington or Marlborough or Nelson or Tasman or “West Coast” or Canterbury or Otago or Southland) |
| S8 | TI (Aborig* or “First People*” or “First Nation” or “First Nations” or Indigenous or Native* or Maori or Iwi or Hapu or Tribe* or Tribal) OR AB (Aborig* or “First People*” or “First Nation” or “First Nations” or Indigenous or Native* or Maori or Iwi or Hapu or Tribe* or Tribal) OR KW (Aborig* or “First People*” or “First Nation” or “First Nations” or Indigenous or Native* or Maori or Iwi or Hapu or Tribe* or Tribal) |
| S9 | (DE "MAORI (New Zealand people) -- Ethnic identity" OR DE "Indigenous Peoples” OR DE “Indigenous Children” OR DE “Indigenous youth") |
| S10 | DE “Health of Indigenous peoples” |
| S11 | S8 OR S9 OR S10 |
| S12 | S7 AND S11 |
| S13 | TI (North America* or “United States” or USA or American) OR AB (North America* or “United States” or USA or American) OR KW (North America* or “United States” or USA or American) |
| S14 | TI (Canada*) OR AB (Canada*) OR KW (Canada*) |
| S15 | S13 OR S14 |
| S16 | TI (Native or Indigenous or “First Nation” or “First Nations” or “First People*” or Tribe* or Tribal or Indian* or Nation or Nations or Inuit* or Aborig*) OR AB (Native or Indigenous or “First Nation” or “First Nations” or “First People*” or Tribe* or Tribal or Indian* or Nation or Nations or Inuit* or Aborig*) OR KW (Native or Indigenous or “First Nation” or “First Nations” or “First People*” or Tribe* or Tribal or Indian* or Nation or Nations or Inuit* or Aborig*) |
| S17 | (DE "FIRST Nations" OR DE "NATIVE Americans" OR DE "ESKIMOS" OR DE "FIRST Nations" OR DE "METIS" OR DE "NATIVE American women" OR DE "OFF-reservation Indians (Native Americans)" OR DE "OLDER Native Americans" OR DE "RESERVATION Indians (Native Americans)") |
| S18 | S16 OR S17 |
| S19 | S15 AND S18 |
| S20 | S6 OR S12 OR S19 |
| S21 | TI (“mobile outreach” OR “mobile outreach clinic” OR “mobile outreach clinics” OR “mobile health” OR “mobile clinic” OR “mobile clinics” OR “mobile health clinic” OR “mobile health clinics” OR “mobile health care clinic” OR “mobile health care clinics” OR “mobile primary health care clinic” OR “mobile health care clinics” OR “mobile health unit*” OR “mobile health care unit*” OR “mobile primary health care unit*” OR “mobile facilit*” OR “mobile health facilit*” OR “mobile health care facilit*” or “mobile primary health care facilit*”) OR AB (“mobile outreach” OR “mobile outreach clinic” OR “mobile outreach clinics” OR “mobile health” OR “mobile clinic” OR “mobile clinics” OR “mobile health clinic” OR “mobile health clinics” OR “mobile health care clinic” OR “mobile health care clinics” OR “mobile primary health care clinic” OR “mobile health care clinics” OR “mobile health unit*” OR “mobile health care unit*” OR “mobile primary health care unit*” OR “mobile facilit*” OR “mobile health facilit*” OR “mobile health care facilit*” or “mobile primary health care facilit*”) OR KW (“mobile outreach” OR “mobile outreach clinic” OR “mobile outreach clinics” OR “mobile health” OR “mobile clinic” OR “mobile clinics” OR “mobile health clinic” OR “mobile health clinics” OR “mobile health care clinic” OR “mobile health care clinics” OR “mobile primary health care clinic” OR “mobile health care clinics” OR “mobile health unit*” OR “mobile health care unit*” OR “mobile primary health care unit*” OR “mobile facilit*” OR “mobile health facilit*” OR “mobile health care facilit*” or “mobile primary health care facilit*”) |
| S22 | TI health N3 (van or bus) OR AB health N3 (van or bus) OR KW health N3 (van or bus) |
| S23 | S21 or S22 |
| S24 | TI (ambulatory N3 service*) OR AB (ambulatory N3 service*) OR KW (ambulatory N3 service*) |
| S25 | DE "OUTPATIENT medical care" |
| S26 | TI (“health service* access” OR “health service* accessibility” OR “health care service* access” OR “health care service* accessibility”) OR AB (“health service* access” OR “health service* accessibility” OR “health care service* access” OR “health care service* accessibility”) OR KW (“health service* access” OR “health service* accessibility” OR “health care service* access” OR “health care service* accessibility”) |
| S27 | (DE "MEDICAL care" OR DE "ADVANCE directives (Medical care)" OR DE "CHILD health services" OR DE "COMMUNITY health services" OR DE "CURATIVE medicine" OR DE "DENTAL care" OR DE "DISCRIMINATION in medical care" OR DE "EMERGENCY medical services" OR DE "HEALTH disparities" OR DE "HEALTH facilities" OR DE "HEALTH self-care" OR DE "HEALTH services accessibility" OR DE "HOSPITAL care" OR DE "HUMANISTIC medicine" OR DE "MANAGED care plans (Medical care)" OR DE "MEDICAL charities" OR DE "MEDICAL compliance" OR DE "MEDICAL screening" OR DE "MENTAL health services" OR DE "OCCUPATIONAL health services" OR DE "OUTPATIENT medical care" OR DE "PATIENT-centered care" OR DE "PRENATAL care" OR DE "PREVENTIVE health services" OR DE "PREVENTIVE medicine" OR DE "PRIMARY health care" OR DE "RURAL health services" OR DE "SCHOOL health services" OR DE "TRANSCULTURAL medical care" OR DE "WOMEN'S health services") |
| S28 | DE "HEALTH promotion" |
| S29 | DE "POPULATION health" |
| S30 | DE "PRIMARY health care" |
| S31 | S26 OR S27 OR S28 OR S29 OR S30 |
| S32 | TI (mobile or mobiles or “wheel based” or travelling or outreach) OR AB (mobile or mobiles or “wheel based” or travelling or outreach) OR KW (mobile or mobiles or “wheel based” or travelling or outreach) |
| S33 | S31 AND S32 |
| S34 | S23 OR S24 OR S33 |
| S35 | S20 AND S34 |
| S36 | TI (disaster or “disaster relief” or “disaster response”) OR AB (disaster or “disaster relief” or “disaster response”) OR KW (disaster or “disaster relief” or “disaster response”) |
| S37 | S35 not S36 |
| S38 | Limiters: Published Date: 20060101-20191231 |

Database(s); **Cochrane Database of Systematic Reviews**

Search Strategy:

(Australia* or Tasmania* or Victoria* or New South Wales* or Queensland* or Northern Territory* or Western Australia* or South Australia*) OR (New Zealand* or Northland or Auckland or Waikato or Bay of Plenty or Gisborne or Hawkes Bay or Taranaki or Whanganui or Manawatu or Wellington or Marlborough or Nelson or Tasman or West Coast or Canterbury or Otago or Southland) OR (Canada) OR (North America* or United States or USA or American) in Title Abstract Keyword AND (Aborig* or First People* or First Nation*1 or Indigenous or Native* or Islander* or "Torres Strait Islander" or Tribe* or Tribal) OR (Oceanic Ancestry Group or Health Services, Indigenous) OR (Native or Indigenous or First Nation*1 or First People* or Tribe* or Tribal or Indian*1 or Nation*1 or Inuit* or Aborig*) in Title Abstract Keyword AND (mobile outreach or mobile outreach clinic*1 or mobile health or mobile clinic*1 or mobile health clinic*1 or mobile health care clinic*1 or mobile primary health care clinic*1 or mobile unit*1 or mobile health unit*1 or mobile health care unit*1 or mobile primary health care unit*1 or mobile facilit*3 or mobile health facilit*3 or mobile health care facilit*3 or mobile primary health care facilit*3) OR (health NEAR/3 van or health NEAR/3 bus) OR (ambulatory health service*1 or ambulatory health care service*1 or ambulatory primary health care service*1) OR (health service*1 access or health service*1 accessibility or health care service*1 access or health care service*1 accessibility) OR (Health promotion or Population health or Primary health care) in Title Abstract Keyword AND (mobile or mobiles or wheel based or travelling or outreach) in Title Abstract Keyword

**Database(s); INFORMIT**

**Search Strategy:**

(TI:Australia* OR TI:Tasmania* OR TI:Victoria* OR TI:"New South Wales" OR TI:Queensland* OR TI:"Northern Territory" OR TI:"Western Australia" OR TI:"South Australia" OR TI:"New Zealand" OR TI:Northland OR TI:Auckland OR TI:Waikato OR TI:"Bay of Plenty" OR TI:Gisborne OR TI:"Hawkes Bay" OR TI:Taranaki OR TI:Whanganui OR TI:Manawatu OR TI:Wellington OR TI:Marlborough OR TI:Nelson OR TI:Tasman OR TI:"West Coast" OR TI:Canterbury OR TI:Otago OR TI:Southland OR TI:Canada OR TI:"North America" OR TI:"United States" OR TI:USA OR TI:American) AND (TI:Aborig* OR TI:"First Nation" OR TI:Indigenous OR TI:Native* OR TI:Islander* OR TI:"Torres Strait Islander" OR TI:Tribe* OR TI:Tribal OR TI:"Oceanic Ancestry Group" OR TI:"Health Services, Indigenous" OR TI:Native OR TI:Indigenous OR TI:"First People" OR TI:Tribe* OR TI:Tribal OR TI:Indian* OR TI:Nation* OR TI:Inuit* OR TI:Aborig*) AND (TI:mobile* OR TI:outreach OR TI:health %3 van OR TI:health %3 bus OR TI:"ambulatory” OR TI:"wheel based" OR TI:travelling) OR (AB:Australia* OR AB:Tasmania* OR AB:Victoria* OR AB:"New South Wales" OR AB:Queensland* OR AB:"Northern Territory" OR AB:"Western Australia" OR AB:"South Australia" OR AB:"New Zealand" OR AB:Northland OR AB:Auckland OR AB:Waikato OR AB:"Bay of Plenty" OR AB:Gisborne OR AB:"Hawkes Bay" OR AB:Taranaki OR AB:Whanganui OR AB:Manawatu OR AB:Wellington OR AB:Marlborough OR AB:Nelson OR AB:Tasman OR AB:"West Coast" OR AB:Canterbury OR AB:Otago OR AB:Southland OR AB:Canada OR AB:"North America*" OR AB:"United States" OR AB:USA OR AB:American) AND (AB:Aborig* OR AB:"First People" OR AB:Indigenous OR AB:Native* OR AB:Islander* OR AB:"Torres Strait Islander" OR AB:Tribe* OR AB:Tribal OR AB:"Oceanic Ancestry Group" OR AB:"Health Services, Indigenous" OR AB:Native OR AB:Indigenous OR AB:"First People" OR AB:Tribe* OR AB:Tribal OR AB:Indian* OR AB:Nation* OR AB:Inuit* OR AB:Aborig* AND (TI:mobile* OR TI:outreach OR TI:health %3 van OR TI:health %3 bus OR TI:"ambulatory” OR TI:"wheel based" OR TI:travelling)
