# Supplementary material for: Mobile primary health care clinics for Indigenous populations in Australia, Canada, New Zealand and the United States: a systematic scoping review
Source: Int J Equity Health. 2020 Nov 9;19:201. doi: 10.1186/s12939-020-01306-0 (PMC7652411; doi:10.1186/s12939-020-01306-0)
Supplement: Supplementary file 3 — Additional file 3. [file 12939_2020_1306_MOESM3_ESM.docx]

**Additional file 3: Grey literature sources searched**

|  | |
| --- | --- |
| **Source** | **URL** |
| Australian and New Zealand Indigenous-specific research | |
| Lowitja Institute | https://www.lowitja.org.au/ |
| Australian Institute of Aboriginal and Torres Strait Islander Studies | https://aiatsis.gov.au/ |
| Indigenous Healthinfonet | https://healthinfonet.ecu.edu.au/ |
| Closing the Gap | https://closingthegap.pmc.gov.au/ |
| National Aboriginal Community Controlled Organization (NACCHO) | https://www.naccho.org.au/ |
| Māori Health Clearinghouse | https://www.health.govt.nz/our-work/populations/maori-health |
| Whakauae Research for Maori Health and Development | https://www.whakauae.co.nz/ |
| United States and Canadian Indigenous-specific research | |
| Indigenous Studies Portal | https://iportal.usask.ca/ |
| Canadian Institute of Indigenous Peoples’ Health | http://www.cihr-irsc.gc.ca/e/8668.html |
| U.S. Department of Health and Human Services Office of Minority Health | https://minorityhealth.hhs.gov/ |
| Centers for American Indian and Alaska Native Health | http://www.ucdenver.edu/academics/colleges/PublicHealth/research/centers/CAIANH/Pages/CAIANH.aspx |
| First Nations Health Authority | http://www.fnha.ca/ |
| Indian Health Service | https://www.ihs.gov/ |
| American Indian and Alaska Native Health | https://americanindianhealth.nlm.nih.gov/ |
| United States and Canadian Indigenous organizations^ | |
| Assembly of First Nations | http://www.afn.ca/ |
| Inuit Tapiriit Kanatami | https://www.itk.ca/ |
| National Congress of American Indians | http://www.ncai.org/ |
| Native Women’s Association of Canada | https://www.nwac.ca/ |
| Alaska Federation of Natives | https://www.nativefederation.org/ |
| North American Youth and Family Center | https://nayapdx.org/about/ |
| Congress of Aboriginal Peoples | http://www.abo-peoples.org/ |
| Métis Nation | http://www.metisnation.ca/ |
| Native Canadian Centre of Toronto | https://ncct.on.ca/ |
| Pauktuutit Inuit Women of Canada | https://www.pauktuutit.ca/ |
| Alaska Native Tribal Health Consortium | https://anthc.org/ |
| Association on American Indian Affairs | https://www.indian-affairs.org/ |
| Administration for Native Americans | https://www.acf.hhs.gov/ana |
| Northwest Portland Area Indian Heath Board | http://www.npaihb.org/ |
| National Indian Council on Aging | https://nicoa.org/ |
| Native American Community Development Institute | https://www.nacdi.org/ |
| Alliance of Colonial Era Tribes | http://www.acet-online.org/ |
| Affiliated Tribes of Northwest Indians | http://www.atnitribes.org/ |
| Tribal Alliance of Sovereign Indian Nations | http://www.usetinc.org/ |
| Rocky Mountain Tribal Leaders Council | https://www.rmtlc.org/ |
| Center for Native American Youth | http://www.cnay.org/ |
| National Council of Urban Indian Health | https://www.ncuih.org/index |
| National Indian Child Welfare Association | https://www.nicwa.org/ |
| National Indian Health Board | http://www.nihb.org/ |
| National Native American EMS Association | http://www.nnaemsa.org/ |
| Indspire | https://indspire.ca/ |
| British Columbia Aboriginal Network on Disability Society | http://www.bcands.bc.ca/links/aboriginal-organizations/ |
| Aboriginal Infant Development Program | http://aidp.bc.ca/ |
| First Nations Health Council | http://fnhc.ca/ |
| National Collaborating Centre for Aboriginal Health | https://www.nccah-ccnsa.ca/en/ |
| Indigenous Physicians Association of Canada | http://www.ipac-amac.ca/ |
| Aboriginal Sexual Health | http://www.aboriginalsexualhealth.ca/index_e.aspx |
| First Nations Child and Family Caring Society | https://fncaringsociety.com/welcome |
| Health services or health research* | |
| Government of Canada | https://www.canada.ca/ |
| National Institutes of Health | https://www.nih.gov/ |
| New Zealand Ministry of Health | https://www.health.govt.nz/ |
| Health Research Council | http://www.hrc.govt.nz/ |
| Department of Health | http://www.health.gov.au/ |
| Australian Institute of Health and Welfare | https://www.aihw.gov.au/ |
| Canadian Institutes of Health Research | http://www.cihr-irsc.gc.ca/ |
| U.S. Department of Health and Human Services | https://www.hhs.gov/ |
| Agency for Healthcare Research and Quality | https://www.ahrq.gov/ |
| Australian Health Services Research Institute | https://ahsri.uow.edu.au/ |
| Canadian Association for Health Services and Policy Research | https://www.cahspr.ca/ |
| World Health Organization | https://www.who.int/ |
| Primary Health Care Research and Information Service (PHCRIS) | http://www.flinders.edu.au/medicine/sites/general-practice/primary-health-care-research-and-information-service/ |
| Rural, remote, or mobile health research or associations* | |
| Mobile Healthcare Association | http://www.mobilehca.org/ |
| Centre for Rural and Northern Health Research | https://www.cranhr.ca/ |
| Services for Australian Rural and Remote Allied Health | https://sarrah.org.au/ |
| Rural Health Information Hub | https://www.ruralhealthinfo.org/ |
| Open access, repositories, or catalogues* | |
| WorldCat | https://www.worldcat.org/ |
| informIT | Through Deakin University |
| Google | http://www.google.com |
| Trove | https://trove.nla.gov.au/ |
| ProQuest Dissertations & Theses Global | Through Deakin University |
| OAIster | https://www.oclc.org/en/oaister.html |

^Indigenous organizations chosen for their focus on health; health education and promotion; children and families; disability; or national leadership or strategic direction

*Online repositories and websites chosen for their comprehensiveness or relevancy to the topic
