# Supplementary material for: Mobile primary health care clinics for Indigenous populations in Australia, Canada, New Zealand and the United States: a systematic scoping review
Source: Int J Equity Health. 2020 Nov 9;19:201. doi: 10.1186/s12939-020-01306-0 (PMC7652411; doi:10.1186/s12939-020-01306-0)
Supplement: Supplementary file 4 — Additional file 4. [file 12939_2020_1306_MOESM4_ESM.docx]

**Additional file 4: Excluded studies**

| **Reference** | **Reason for exclusion** |
| --- | --- |
| Agostini J, Heazlewood R, Ruben A. Cape York Paediatric Outreach Clinic - Improving access to primary care in the Cape York Peninsula region. Australian Family Physician. 2012;41(8):623-625 | No mobile clinic |
| Abdul Halim M, Abdual Halim M, Abdul Halim D, Goodman A et al. Characteristics of Lipid Profiles in Indigenous Cardiac Outreach Program Cohort in Rural and Remote Communities in Queensland: A Snapshot After 10 years of Service. Heart Lung and Circulation. 2017;26 Suppl 2:S330. | No mobile clinic |
| Abdul Halim M, Goodman A, Corpus R & McKenzie S. The Demographics and Natural History of Rheumatic Heart Disease: A Look into Indigenous Cardiac Outreach Programme Cohort after Nearly a Decade of Service. Heart Lung and Circulation. 2017;26:S337. | No mobile clinic |
| Allan J. Engaging primary health care workers in drug and alcohol and mental health interventions: challenges for service delivery in rural and remote Australia. Australian Journal of Primary Health. 2010;16(4):311-318 | No mobile clinic |
| Allison MT, Rivers PA, Fottler MD. Future public health delivery models for Native American tribes. Public Health. 2007;121(4):296-307. | No mobile clinic |
| Anderson S. Management of indigenous chronic middle ear disease in North Queensland, Australia. Journal of Laryngology and Otology. 2016;130:S26. | No mobile clinic |
| Anonymous. Increase mobile nurse clinics. Australian Nursing Journal. 2010;18(5):9-9. | No mobile clinic |
| Askew DA, Togni SJ, Schluter PJ, Rogers L et al. Investigating the feasibility, acceptability and appropriateness of outreach case management in an urban Aboriginal and Torres Strait Islander primary health care service: a mixed methods exploratory study. BMC Health Services Research. 2016;16(1):1-14. | No mobile clinic |
| Australian Institute of Health and Welfare. Hearing health outreach services to Aboriginal and Torres Strait Islander children and young people in the Northern Territory: 2012–13 to 2014–15 [Internet]. Canberra; Australian Institute of Health and Welfare: 2015. Available from: https://www.aihw.gov.au/reports/indigenous-australians/hearing-health-outreach-services-2012-13-2014-15/contents/table-of-contents. Accessed 10 Jan 2020. | No mobile clinic |
| Australian Institute of Health and Welfare. Hearing health outreach services to Indigenous children and young people in the Northern Territory: 2012–13 and 2013–14 [Internet]. Canberra; Australian Institute of Health and Welfare: 2015. Available from: https://www.aihw.gov.au/reports/indigenous-australians/hearing-health-outreach-services-2012-13-2013-15/contents/table-of-contents. Accessed 10 Jan 2020. | No mobile clinic |
| Bowen A, Muhajarine N. Prevalence of antenatal depression in women enrolled in an outreach program in Canada. Journal of Obstetrics, Gynecologic and Neonatal Nursing. 2006;35(4):491-498. | No mobile clinic |
| Brown M, Wigg N, Turner K. Ear disease and Indigenous kids: tackling the silent epidemic. Journal of Paediatrics and Child Health. 2011;47(1):18-19. | No mobile clinic |
| National Center for Chronic Disease Prevention and Health Promotion. Good Health and Wellness in Indian Country [Internet]. National Center for Chronic Disease Prevention and Health Promotion: 2016. https://www.cdc.gov/chronicdisease/pdf/GHWIC-AAG.pdf. Accessed 5 Jan 2020. | No mobile clinic |
| Corpus RJ, Laakso T. Can Indigenous cardiac health "close the gap" through a state-wide capacity building approach? Cardiology in the Young. 2010;20 Suppl 1:1-422. | No mobile clinic |
| Cresp et al. Effectiveness of the Koorliny Moort out-of-hospital health care program for Aboriginal and Torres Strait Islander children in Western Australia. Medical Journal of Australia. 2016;204(4):1971-1977. | No mobile clinic |
| Griffin S, Layton B. Bringing care closer to home. Canadian Nurse. 2008;104(6):12-13. | No mobile clinic |
| Hurley R, Felland L, Lauer J. Community health centers tackle rising demands and expectations. Center for Studying Health System Change. 2007;116(1):1-4. | No mobile clinic |
| Kok et al. Hearing health outreach services to Aboriginal and Torres Strait Islander children and young people in Northern Territory:2012-13 to 2014-15 [Internet]. Canberra; Australian Institute of Health and Welfare: 2015. Available from: https://www.aihw.gov.au/getmedia/c19d5089-6640-4a86-a87c-00f578e7b849/19422.pdf.aspx?inline=true. Accessed 7 Jan 2020. | No mobile clinic |
| Ministry of Health New Zealand. Closer to home [Internet]. Wellington; Ministry of Health New Zealand: 2019. Available from. https://www.health.govt.nz/new-zealand-health-system/new-zealand-health-strategy-future-direction/five-strategic-themes/closer-home. Accessed 5 Jan 2020. | No mobile clinic |
| Ministry of Health New Zealand. National Review of Outreach Immunisation Services: Summary and Recommendations [Internet]. Wellington; Ministry of Health New Zealand: 2016. Available from: https://www.health.govt.nz/publication/national-review-outreach-immunisation-services-summary-and-recommendations. Accessed 6 Jan 2020. | No mobile clinic |
| Redwood D, Provost E, Lopez ED, Skewes M et al. A Process Evaluation of the Alaska Native Colorectal Cancer Family Outreach Program. Health Education & Behaviour. 2016;43(1):35-42. | No mobile clinic |
| Roubidoux M. Breast cancer and screening in American Indian and Alaska Native women. Journal of Cancer Education. 2012;27(1):S66-72. | No mobile clinic |
| Tibby D, Corpus R. Innovative model of cardiac care reaches out to remote Queensland. The Indigenous Outreach Program. 2008;27(2):10-11. | No mobile clinic |
| Bryce et al. Key Principles of Urban Indigenous Cardiac Outreach Clinics. Heart Lung and Circulation. 2019;28(1):S63. | No mobile clinic |
| Tuttle et al. Overcoming the tyranny of distance: An analysis of outreach visits to optimise secondary prevention of cardiovascular disease in high-risk individual living in Central Australia. Australian Journal of Rural Health. 2016;24(2):99-105. | No mobile clinic |
| Archibald-Binge E. A vision for Palm: Mobile eye clinic saves sight in First Nations communities [Internet]. Living Black: 2017. Available from: https://www.sbs.com.au/nitv/living-black/article/2017/02/22/vision-palm-mobile-eye-clinic-saves-sight-first-nations-communities. Accessed 4 Jan 2020. | Not a primary health care mobile clinic |
| Bax K, Shedda S, Frizelle F. The New Zealand mobile surgical bus service: what is it achieving. The New Zealand Medical Journal. 2006;119(1236):U2025. | Not a primary health care mobile clinic |
| Belardi L. Mobile dialysis gives patients a ticket home. Nursing Review. 2012:16-17 | Not a primary health care mobile clinic |
| Conway J, Lawn S, Crail S, McDonald S. Indigenous patient experiences of returning to country: a qualitative evaluation on the Country Health SA Dialysis bus. BMC Health Services Research. 2018;18(1010):1-13. | Not a primary health care mobile clinic |
| Copeland S, Muir J, Turner A. Understanding Indigenous patient attendance: A qualitative study. Australian Journal of Rural Health. 2017;25(5):268-274. | Not a primary health care mobile clinic |
| Diao et al. The Effectiveness of a Mobile Clinic in Improving Follow-up Eye Care for At-Risk Children. Journal of Paediatric Opthalmology. 2016;53(6):344-348. | Not a primary health care mobile clinic |
| Dobson L, Tan I, Wu XN, Muir J et al. Lions outback vision van- 3 month preliminary audit of service to rural and remote Western Australia. Clinical and Experimental Ophthalmology. 2016;44:98. | Not a primary health care mobile clinic |
| Fraser Coast Chronicle. Clinic on wheels is vital for Indigenous eye treatment [Internet]. Fraser Coast Chronicle: 2014. Available from: https://www.frasercoastchronicle.com.au/news/clinic-is-a-vital-facility/2161712/. Accessed 10 Jan 2020. | Not a primary health care mobile clinic |
| Kidney Health Australia. Big Red Kidney Bus [Internet]. South Melbourne; Kidney Health Australia: 2020. Available from: https://kidney.org.au/bus. Accessed 10 Jan 2020. | Not a primary health care mobile clinic |
| Lions Outback Vision. Vision Van [Internet]. Nedlands; Lions Outback Vision: 2020. Available from: https://www.outbackvision.com.au/vision-van/. Accessed 11 Jan 2020. | Not a primary health care mobile clinic |
| Ritchie P. Mobile eye clinic a hit at Indigenous facilities. Optometry Australia: 2018. Available from: https://www.optometry.org.au/workplace/mobile-eye-clinic-a-hit-at-indigenous-facilities/. Accessed 13 Jan 2020. | Not a primary health care mobile clinic |
| Shashibhaskara S, Cherian S, Pawar B, Fernandes D et al. Respite hemodialysis to remote Indigenous central Australian population and mobile dialysis unit. Nephrology. 2012;17:47. | Not a primary health care mobile clinic |
| Anonymous. Queensland mobile women's health service turns 21. Australian Nursing Journal. 2012;19(11):43. | Not an Indigenous-specific mobile clinic |
| Arnold-Reed D, Troeung L, Brett T, Chan She Ping-Delfos W et al. Increasing multimorbidity in an Australian street health service: A 10-year retrospective cohort study. Australian Journal of General Practice. 2018;47(4):181-189. | Not an Indigenous-specific mobile clinic |
| Aslam F, Foody J & Verma B. Prevalence of hypertension in urban Boston female population: A family van mobile health model. Journal of Clinical Hypertension. 2009;11(4):A14. | Not an Indigenous-specific mobile clinic |
| Atkins E, Madhavan S, LeMasters T, Vyas A et al. Are Obese Women More Likely to Participate in a Mobile Mammography Program?. Journal of Community Health. 2013;38(2):338-348. | Not an Indigenous-specific mobile clinic |
| Australian Institute of Health and Welfare (AIHW). Breast screening program reaching target group [Internet]. Canberra; Australian Institute of Health and Welfare: 2012. Available at: https://www.aihw.gov.au/news-media/media-releases/2012/2012-oct/breast-screening-program-reaching-target-group. Accessed 14 Jan 2020. | Not an Indigenous-specific mobile clinic |
| Brooks SE, Hembree TM & Dignan MB. Mobile Mammography in Underserved Populations: Analysis of Outcomes of 3,923 Women. Journal of Community Health. 2013;38(5):900-906. | Not an Indigenous-specific mobile clinic |
| Chen Y, Chang-Halpenny C & Braddock C. Perspectives of Mobile Versus Fixed Mammography in Santa Clara County, California: A Focus Group Study. Cureaus. 2016;8(2). | Not an Indigenous-specific mobile clinic |
| Dawkins E, Michimi A, English G. Dental caries among children visiting a mobile dental clinic in South Central Kentucky: a pooled cross-sectional study. BMC Oral Health. 2013;13(19). | Not an Indigenous-specific mobile clinic |
| Doctors of the World. Mobile Health Clinic Montreal [Internet]. Québec; Doctors of the World: 2020. Available from: https://www.medecinsdumonde.ca/en/action-mdm/mobile-clinic/. Accessed 15 Jan 2020. | Not an Indigenous-specific mobile clinic |
| Drake B, Salmafatima S, Lyons S et al. Mammograms On-the-Go- Predictors of Repeat Visits to Mobile Mammography Vans in St Louis, Missouri, USA: A Case-Control Study. BMJ Open 2015;5(3). | Not an Indigenous-specific mobile clinic |
| Eagle Feather News. Mobile Health Unit Will Serve Core Neighbourhoods [Internet]. Eagle Feather News: 2008. Available from: https://www.eaglefeathernews.com/quadrant/media//pastIssues/Sept_2008.pdf. Accessed 15 Jan 2020. | Not an Indigenous-specific mobile clinic |
| Finley J, Webb B. Use of Mobile Health Clinics (MHC) to Detect and Reduce Hypertension among Patients in East Los Angeles. Brandman University: 2019 | Not an Indigenous-specific mobile clinic |
| Gardner T, Gavaza P, Meade P, Adkins D. Delivering Free Healthcare to Rural Central Appalachia Population: The Case of the Health Wagon. Rural and Remote Health. 2012;12(1). | Not an Indigenous-specific mobile clinic |
| Gibson B, Debarchana G, Altice F. Accessibility and utilization patterns of a mobile medical clinic among vulnerable populations. Health Place. 2014;28:153-66. | Not an Indigenous-specific mobile clinic |
| Agency for Healthcare Research and Quality. Going the Extra Mile to Provide Community-based Care [Internet]. Rockville; Agency for Healthcare Research and Quality: 2018. Available from: https://www.ahrq.gov/evidencenow/about/stories/profiles/st-josephs.html. Accessed 16 Jan 2020. | Not an Indigenous-specific mobile clinic |
| Goldberg K. Mobile Medical Units Deliver To San Diego's Underserved [Internet]. KPBS news: 2014. Available from: https://www.kpbs.org/news/2014/jun/17/mobile-medical-units-deliver-underserved/. Accessed 17 Jan 2020. | Not an Indigenous-specific mobile clinic |
| Hamill S. Pittsburgh Mercy rolls out mobile health care [Internet]. Gazette: 2014. Available from: https://www.post-gazette.com/local/region/2014/10/13/Pittsburgh-Mercy-rolls-out-mobile-health-care/stories/201410100027. Accessed 18 Jan 2020. | Not an Indigenous-specific mobile clinic |
| Helseth C. The Health Wagon Serves Up Care to Virginia's Rural Uninsured [Internet]. Rural Health Information: 2009. Available from: https://www.ruralhealthinfo.org/rural-monitor/health-wagon-serves-up-care/. Accessed 10 Jan 2020. | Not an Indigenous-specific mobile clinic |
| Hill C, Zurakowski D & Oriol N. Knowledgeable Neighbours: A Mobile Clinic Model for Disease Prevention and Screening in Undeserved Communities. American Journal of Public Health. 2012;102(3):406-410. | Not an Indigenous-specific mobile clinic |
| Langelier M, Moore J, Carter R, Boyd L, Rodat C. An assessment of Mobile and Portable Dentistry Programs to Improve Population Oral Health [Internet]. New York; Oral Health Workforce Research Center: 2017. Available from: https://www.chwsny.org/wp-content/uploads/2017/09/OHWRC_Mobile_and_Portable_Dentistry_Programs_2017.pdf. Accessed 10 Jan 2020. | Not an Indigenous-specific mobile clinic |
| NIH Record. VRC Launches Mobile Clinic [Internet]. NIH Record: 2008. Available from: https://nihrecord.nih.gov/sites/recordNIH/files/pdf/2008/NIH-Record-2008-07-25.pdf. Accessed 10 Jan 2020. | Not an Indigenous-specific mobile clinic |
| Oriol NE, Cote PJ & Kohane I. Calculating the return on investment of mobile healthcare. BMC Medicine. 2009;7(27):1-6. | Not an Indigenous-specific mobile clinic |
| Prairie Mountain Health. Mobile Clinic [Internet]. Manitoba; Prairie Mountain Health: 2019. Available from: https://www.prairiemountainhealth.ca/mobile-clinic. Accessed 13 Jan 2020. | Not an Indigenous-specific mobile clinic |
| Rural Health Information Hub. Delta Dental Mobile Program [Internet]. Grand Forks; Rural Health Information Hub: 2020. Available from: https://www.ruralhealthinfo.org/project-examples/626. Accessed 15 Jan 2020. | Not an Indigenous-specific mobile clinic |
| Rural Health Information Hub. Miles for Smiles Mobile Dental Unit [Internet]. Grand Forks; Rural Health Information Hub: 2020. Available from: https://www.ruralhealthinfo.org/project-examples/531. Accessed 16 Jan 2020. | Not an Indigenous-specific mobile clinic |
| Rural Health Information Hub. Mobile Clinics - Rural Transportation Toolkit. Grand Forks; Rural Health Information Hub: 2020. Available from: https://www.ruralhealthinfo.org/toolkits/transportation/2/models-to-overcome-barriers/mobile-clinics. Accessed 17 Jan 2020. | Not an Indigenous-specific mobile clinic |
| Rural Health Information Hub. Mobile Dental Services Model [Internet]. Grand Forks; Rural Health Information Hub: 2020. Available from: https://www.ruralhealthinfo.org/toolkits/oral-health/2/mobile-dental-services-model. Accessed 19 Jan 2020. | Not an Indigenous-specific mobile clinic |
| Rural Health Information Hub. Mobile Services in Florida Helps Homeless Veterans [Internet]. Grand Forks; Rural Health Information Hub: 2020. Available from: https://www.ruralhealthinfo.org/rural-monitor/mobile-service-helps-homeless-veterans/. Accessed 17 Jan 2020. | Not an Indigenous-specific mobile clinic |
| Rural Health Information Hub. Mobile Unit Model [Internet]. Grand Forks; Rural Health Information Hub: 2020. Available from: https://www.ruralhealthinfo.org/toolkits/services-integration/2/care-coordination/mobile-unit. Accessed 14 Jan 2020. | Not an Indigenous-specific mobile clinic |
| Rural Health Information Hub. The Health Wagon [Internet]. Grand Forks; Rural Health Information Hub: 2020. Available from: https://www.ruralhealthinfo.org/project-examples/711. Accessed 10 Jan 2020. | Not an Indigenous-specific mobile clinic |
| Roth R, Newhouse R, Robinson B, Faulkner S & Remick SC. Bonnie's Bus --cancer disparities in West Virginia, philanthropy and opportunities to build lasting partnerships. The West Virginia Medical Journal. 2009;105:68-72. | Not an Indigenous-specific mobile clinic |
| Shank J. The Mobile Health Clinic That's Been Helping The Poor For Over 40 years [Internet]. Nation Swell: 2014. Available from: http://nationswell.com/mobile-health-program-helping-arizona-poor/. Accessed 9 Jan 2020. | Not an Indigenous-specific mobile clinic |
| Smeltz A. Clinics go mobile to bring health care to streets of Western Pennsylvania [Internet]. Trib Live: 2014. Available from: https://archive.triblive.com/news/clinics-go-mobile-to-bring-health-care-to-streets-of-western-pennsylvania/. Accessed 9 Jan 2020. | Not an Indigenous-specific mobile clinic |
| Song Z, Hill C & Oriol NE. Mobile Clinic in Massachusetts Associated With Cost Savings From Lowering Blood Pressure And Emergency Department Use. Health Affairs. 2013;32(1):36-44. | Not an Indigenous-specific mobile clinic |
| South Dakota Cancer Coalition. Environmental Scan of South Dakota's Mobile Mammography Efforts [Internet]. South Dakota Cancer Coalition: 2019. Available from: http://sdaho.org/wp-content/uploads/2019/05/Final-Environmental-Scan-of-SD-Mobile-Mammography-Services.pdf. Accessed 7 Jan 2020. | Not an Indigenous-specific mobile clinic |
| Times Free Press. New Mobile Clinic Brings HIV, Sexually Transmitted Infection Treatment to Rural Communities Across South East Tennessee [Internet]. Times Free Press: 2019. Available from: https://www.timesfreepress.com/news/local/story/2019/jul/31/new-mobile-clinic-brings-hiv-sti-treatment-ru/500218/. Accessed 7 Jan 2020. | Not an Indigenous-specific mobile clinic |
| United States Department of Health and Human Services. HHS Office of Minority Health announces new research study of mobile health clinics [Internet]. United States Department of Health and Human Services: 2011. Available from: https://minorityhealth.hhs.gov/omh/content.aspx?ID=9221. Accessed 8 Jan 2020. | Not an Indigenous-specific mobile clinic |
| United States Department of Health and Human Services. TAG in Action: Mobile Teen Van [Internet]. Youth.Gov: 2020. Available from: https://www.hhs.gov/ash/oah/tag/in-action/mobile-health-van/index.html. Accessed 15 Jan 2020. | Not an Indigenous-specific mobile clinic |
| Zimmerman R. Study: Mobile Clinic Saves Money, Improves Health For Low Income Patients. 2013. Available from: https://www.wbur.org/commonhealth/2013/01/15/mobile-health-clinic-saves-money. Accessed 18 Jan 2020. | Not an Indigenous-specific mobile clinic |
| Vang S, Margolies LR, Jandorf L. Mobile mammography participation among medically underserved women: a systematic review. Preventing Chronic Disease. 2018;15:180291. | Sub-studies already included in search |
| Vashishtha V, Kote S, Ravneet KM. Reach the Unreached - A Systematic Review on Mobile Dental Units. Journal of Clinical Diagnostic Research. 2014;8(8):5-8. | Sub-studies already included in search |
| Yu et al. The scope and impact of mobile health clinics in the United States: a literature review. International Journal for Equity in Health. 2017;16:1:12. | Sub-studies already included in search |
| Abdel-Aleem H, El-Gibaly O, El-Gazzar A, Al-Attar GST. Mobile clinics for women's and children's health. Cochrane Database of Systematic Reviews. 2016. doi: https://doi.org/10.1002/14651858.CD009677.pub2 | Sub-studies did not meet inclusion criteria |
| Hill CF, Powers BW, Jain SH, Bennet J et al. Mobile health clinics in the era of reform. American Journal of Management Care. 2014;20(3):261-264. | Sub-studies did not meet inclusion criteria |
| Huffman MD & Galloway JM. Cardiovascular Health in Indigenous Communities: Successful Programs. Heart Lung and Circulation. 2010;19(5-6):351-360. | Sub-studies did not meet inclusion criteria |
| Curnow, D. Queensland Health Mobile Launched: A $1.5 million health screening van has been launched to fight medical problems in Indigenous communities across Queensland [podcast]. Queensland; ABC radio: 2008. | Audio-recording not available |
| Network for Indigenous Cultural and Health Education. The Deadly Ears Mobile Van [podcast]. NICHE. 2015. Accessed 2020 8 June. Available from: http://nicheportal.org/the-deadly-ears-mobile-van-2/. Accessed 17 Jan 2020. | Audio-recording not available |
